# Differential expression of transposable elements in the medaka melanoma model

**DOI:** 10.1371/journal.pone.0251713

**Published:** 2021-10-27

**Authors:** Frederik Helmprobst, Susanne Kneitz, Barbara Klotz, Magali Naville, Corentin Dechaud, Jean-Nicolas Volff, Manfred Schartl

**Affiliations:** 1 Physiological Chemistry, Biocenter, University of Würzburg, Würzburg, Germany; 2 Department of Neuropathology, Philipps-University Marburg, Marburg, Germany; 3 Biochemistry and Cell Biology, Biocenter, University of Würzburg, Würzburg, Germany; 4 Institut de Génomique Fonctionnelle de Lyon, Ecole Normale Supérieure de Lyon, Université Lyon, Lyon, France; 5 The Xiphophorus Genetic Stock Center, Department of Chemistry and Biochemistry, Texas State University, San Marcos, Texas, United States of America; 6 Developmental Biochemistry, University of Würzburg, Würzburg, Germany; University of Muenster, GERMANY

## Abstract

Malignant melanoma incidence is rising worldwide. Its treatment in an advanced state is difficult, and the prognosis of this severe disease is still very poor. One major source of these difficulties is the high rate of metastasis and increased genomic instability leading to a high mutation rate and the development of resistance against therapeutic approaches. Here we investigate as one source of genomic instability the contribution of activation of transposable elements (TEs) within the tumor. We used the well-established medaka melanoma model and RNA-sequencing to investigate the differential expression of TEs in wildtype and transgenic fish carrying melanoma. We constructed a medaka-specific TE sequence library and identified TE sequences that were specifically upregulated in tumors. Validation by qRT- PCR confirmed a specific upregulation of a LINE and an LTR element in malignant melanomas of transgenic fish.

## Introduction

Worldwide incidence of melanoma has steadily increased over the last decades [1] and prognosis of this most aggressive form of skin cancer is still very poor due its high metastatic potential. Therapeutic treatments when the disease is already in its progression state is difficult and one great problem is the genomic instability of these tumors [2]. Genomic instability leads to mutations, including chromosome structure rearrangements, point mutations, and microsatellite instability and other smaller structure variations within the genetic code. These mutations can result in bypassing intra- and extracellular control systems, giving cancerous cells a growth advantage and inducing further selection towards higher malignancy [3].

Due to the high abundance (~45%) of transposable elements (TEs) in the human genome [4], they can be one major source of genomic instability and can lead, when activated, to further mutational changes within the genome. These changes may contribute to the increasing resistance of some cells against drugs during melanoma treatment and lead to further progression of the disease [5]. TEs, normally silenced by DNA methylation [6], can be activated by the common loss of the epigenetic regulation as found in human cancers [7–12]. It was also shown that regulatory sequences in promoter regions derived from TEs [13, 14] have important functions in human gene expression [15]. Changes in the methylation pattern within these sequences can lead to an overexpression of proto-oncogenes, like CSF1R [16] or FABP7 [17, 18].

TEs can be classified [19] due to their transposition mechanism into two categories. Retrotransposons (class I) propagate via an RNA intermediate by “copying and pasting” the element at a different genomic position. Class II elements, or DNA transposons, change their position via a DNA intermediate by “cutting and pasting” from one genomic locus to another. Class I TEs can be further divided in different subgroups. One group corresponds to the Long Terminal Repeat-retrotransposons (LTR) [20]. These TEs encode several proteins including a protease, a group-specific antigen (Gag), and a polymerase (Pol), which has reverse transcriptase, integrase and RNase activity [21]. In vertebrates, four families of LTR retrotransposons are present, including the *Gypsy/Ty3*, *Ty1/Copia*, and *BEL/Pao* families, and the more divergent *DIRS* transposons [22, 23]. Another family of TEs possesses a structure similar to LTR retrotransposons, but with an additional envelope (Env)-like protein encoded within their sequence, which they share with viral relatives. This feature motivated to call them endogenous retroviruses [21, 24]. The second group is constituted by the non-LTR retrotransposons, which include long and short interspersed elements (LINEs and SINEs) [25]. SINEs can further be divided into several families [26]. LINEs have one or two open reading frames (ORF), which encodes for a protein with RNA binding activity, and a protein with an endonuclease and reverse transcriptase domain [27, 28]. In all TE classes autonomous and non-autonomous elements can be found. LINEs represent the autonomous non-LTR retrotransposons, which can propagate on their own, by a specific RNA sequence in their 3’-tail, which they can share with SINE elements or, in the case of the L1 transposon via a polyA-tailed RNA intermediate [29, 30]. The non-autonomous SINEs such as Alu elements cannot spread on their own, but can be mobilized by autonomous TEs [29, 30]. The mechanisms of the autonomous transposition of LTR TEs is well described for the Tf1 LTR transposon of *Schizosaccharomyces pombe*, which belongs to the *Gypsy/Ty3* family. It is transcribed in a polyA-tailed RNA strand and later reverse transcribed in a virus like particle [31]. In addition, several endogenous retroviruses were discovered in whole transcriptome data from polyA-enriched RNA of human brain [32]. Among vertebrate genomes, the amount, composition and activity of TEs can vary widely. While In humans ~45% of the genome is TE derived, in medaka, the TE content is only around 30% [4, 22, 33]. Most of the TEs in the medaka genome are either DNA or unclassified TEs, whereas the amount of retrotransposons, like LTR, LINE and SINE elements, is much higher in human and comprises nearly 40% of the whole genome [22]. While in humans only one major active autonomous element was discovered so far, namely LINE1 [34], in the small teleost fish medaka several active TEs, for example *Tol1* and *Tol2* elements, were found [25, 35].

To investigate a possible role of TEs in cancer development, we used the well-established medaka melanoma model [36–38]. In these fish, tumor development is induced by the transgenic expression of the *xmrk* oncogene, derived from *Xiphophorus maculatus*, under the control of the pigment cell-specific *mitfa* promoter [38]. The expression of *xmrk* leads to the development of aggressive and highly invasive melanoma, with a great anatomical, molecular and genetical similarity between human and medaka melanomas [38–41].

The purpose of this study was to investigate the expression profile of Class I TEs in wildtype and melanoma developing medaka. For this approach we generated a medaka-specific TE sequence library to scrutinize previously generated RNA-seq data [41, 42]. The RNA-seq data were mapped to the medaka specific TE library and analyzed for differentially expressed TEs. Further validation by qRT-PCR and characterization of the elements revealed increased levels of transcripts of one LINE and one LTR TE family in malignant melanoma indicative of a tumor-specific higher activity that could contribute to a higher genomic instability in melanoma.

## Material methods

### Fish maintenance

Transgenic medaka (*Oryzias latipes*) of the Carbio *tg(mitfa*:*xmrk)* strain [38] and wildtype medaka (Carbio strain) were kept under standard conditions at 25°C water temperature, a light-dark cycle of 14 h light/10 h darkness and were raised in accordance with established protocols [43]. All experiments were performed according to the guidelines of the German animal welfare law and approved by the Government of Lower Franconia (Tierschutzgesetz §11, Abs. 1, Nr. 1, husbandry permit number 568/300-1870/13).

### Construction of the species-specific repeat library

An initial repeat library was obtained using RepeatModeler (Smit, AFA, Hubley, R; http://www.repeatmasker.org) with default parameters. This sequence collection was then refined by the following procedure. Short consensus repeats (<80 nts) were removed. Satellite sequences as well as putative DNA or LTR transposable elements (TEs) were reannotated by aligning each consensus against itself, which allows to visualize internal repeats. BlastX [44] was used to blast «unknown» repeats against the NCBI database, which allowed the removal of multigene families erroneously identified as putative transposable elements. BlastN of the sequence library against itself was applied to remove redundant consensi (e-value < 1e-20 and alignment on at least 80% of both sequences length). In addition, BlastX of «unknown» repeats against an in-house fish transposable element protein library was used to rename matching “unknown” elements according to their hits [22]. Two helitron elements identified by the program HelitronScanner [45] and gypsy, erv1 and copia elements identified by the program LTRharvest [46] that were not identified previously by RepeatModeler were added to the library. Finally, sequences of the library predicted to correspond to SINEs according to the SINE-scan program [47] were reannotated.

### Localization of TEs in the genome

The TE library built in the previous step was used as repeat database for a RepeatMasker search in the genome (Smit, AFA, Hubley, R; http://www.repeatmasker.org). Overlaps in RepeatMasker output were discarded by selecting highest scoring elements. Repeat fragments closer than 20 bp and having the same name were merged.

### TE landscape

For each TE family, all genomic insertions were retrieved and aligned together using Mafft [48]. Global DNA sequence identity was then computed for each possible pair of sequences, excluding gaps. The landscape graph was drawn by reporting the total number of pairwise comparisons for a given family to the total genomic density of this family.

### RNA-seq data and mapping on TE

RNA seq data previously generated in our lab from wildtype and tg (*mitfa*:*xmrk*) medaka larvae [41, 42] were used (Accession number: PRJNA717153). The reads were mapped against our medaka specific TE library using the Burrows-Wheeler Aligner algorithm mem (BWA, http://bio-bwa.sourceforge.net/) allowing multimapping of reads. For reads with an equal score in multiple locations, one of the locations is chosen at random. To get the genomic locations (Oryzias_latipes.ASM223467v1.102) of the reads which mapped to the selected TEs, a fastq file was built from the alignment files obtained in the first step. Next, the BWA alignment algorithm bwasw was used with the threshold allowed for multimapping reads < 1000 (-z 999). Single reads with no adjacent reads within 1kb were removed. For the annotation of genomic positions of the reads we used HOMER (http://homer.ucsd.edu/homer/ngs/annotation.html). Differential expression was calculated with DESeq2 [49].

### TE domain characterization

To obtain full length TEs, we used the tool consensus2genome **(**https://github.com/clemgoub/consensus2genome**).** For characterization of TE structure the sequences from our TE library were analyzed with NCBI domain finder [50, 51]. The TE localization within the Medaka genome (ASM223467v1) were found as previously described with the Blast software [44] by using the consensus sequence of identified differentially expressed TE family as query and visualized with Circoletto [52]. The colour scoring was adjusted within the script. The ration of the blast score divided through the maximal blast score was changed to blue< = 0.25, green< = 0.70, orange< = 0.90, and red>0.90. The amount of ribbons which were allowed to untangle during the process was set to higher (600).

### RNA-seq validation and qPCR

RNA-seq data were confirmed by qPCR on cDNA extracted from whole body of juvenile control and *tg(mitfa*:*xmrk)* larvae. In addition, to study the expression in healthy organs and melanoma, several tissues were extracted from adult fish. RNA was extracted as follows [24]. 1 μg RNA was treated with DNAse I (Thermo Scientific) and transcribed into cDNA using the RevertAid First Strand cDNA Synthesis kit (Thermo Scientific) with random hexamer primers. An amount of 25 ng cDNA of each sample was analyzed in duplicate in a 25 μl reaction volume using a SYBR green-containing mastermix. For qPCR, a mastercycler EP realplex (Eppendorf, Hamburg Germany) was used with 5 min at 95°C followed by 40 cycles of 95°C for 30 s, 60°C for 30 s and 72°C for 20 s. PCR primer sequences are listed in Table 1. Expression of each gene was normalized to efa1 levels. Relative expression was calculated with the 2^-dCT method [53] and the p-values were calculated by Mann-Whitney U-tests.

**Table 1:** Sequences of qRT-PCR primers.

Olat_rnd-5_family-280#LINE/I_for	GAGGGAAATGAAATGGCTGA
Olat_rnd-5_family-280#LINE/I_rev	AACCAGTGTGTCCCATCCTC
Olat_copia_12#LTR/Copia_for	TCTCGATCGATGGGTGCATG
Olat_copia_12#LTR/Copia_rev	GGGAGGTAGGTGGGTGTACT
Olat_gypsy_138#LTR/Gypsy_for	TCTTTGTGGGGAAGCGAGAC
Olat_gypsy_138#LTR/Gypsy_rev	AAACGCCGTCTTCCACTCAT
efa1_for	GCCCCTGGACACAGAGACTTCAT
efa1_rev	AAGGGGGCTCGGTGGAGTCCAT

## Results

### Medaka TE landscape

A specific library of *o*ver 4000 repeat sequences (S1 File) was build using a combination of *de novo* prediction tools. This library was then used as input for RepeatMasker to localize TE sequences in the genome. In total, TEs cover 33.6% of the medaka genome, and mainly comprise DNA and LINE elements (45.3% and 34.7% of the whole TE coverage, respectively, Table 2). This amount of TEs is in the range of the transposon content reported in other studies for the medaka organism [33].

**Table 2:** TE coverage in the genome of *O*. *latipes*.

Class	Coverage (Mb)	% of genome	% of TEs
DNA	111.7	15.2	45.3
RC	0.9	0.12	0.4
LINE	85.5	11.6	34.7
SINE	6.1	0.82	2.5
Retro non-LTR	0.03	0.005	0.01
LTR	30.4	4.1	12.3
Retro unclassified	6.2	0.84	2.5
Unknown	5.8	0.8	2.4
Total	346.6	33.6	

### Expressed transposons in wt and *tg(mitfa*:*xmrk)*

**Fig 1:**
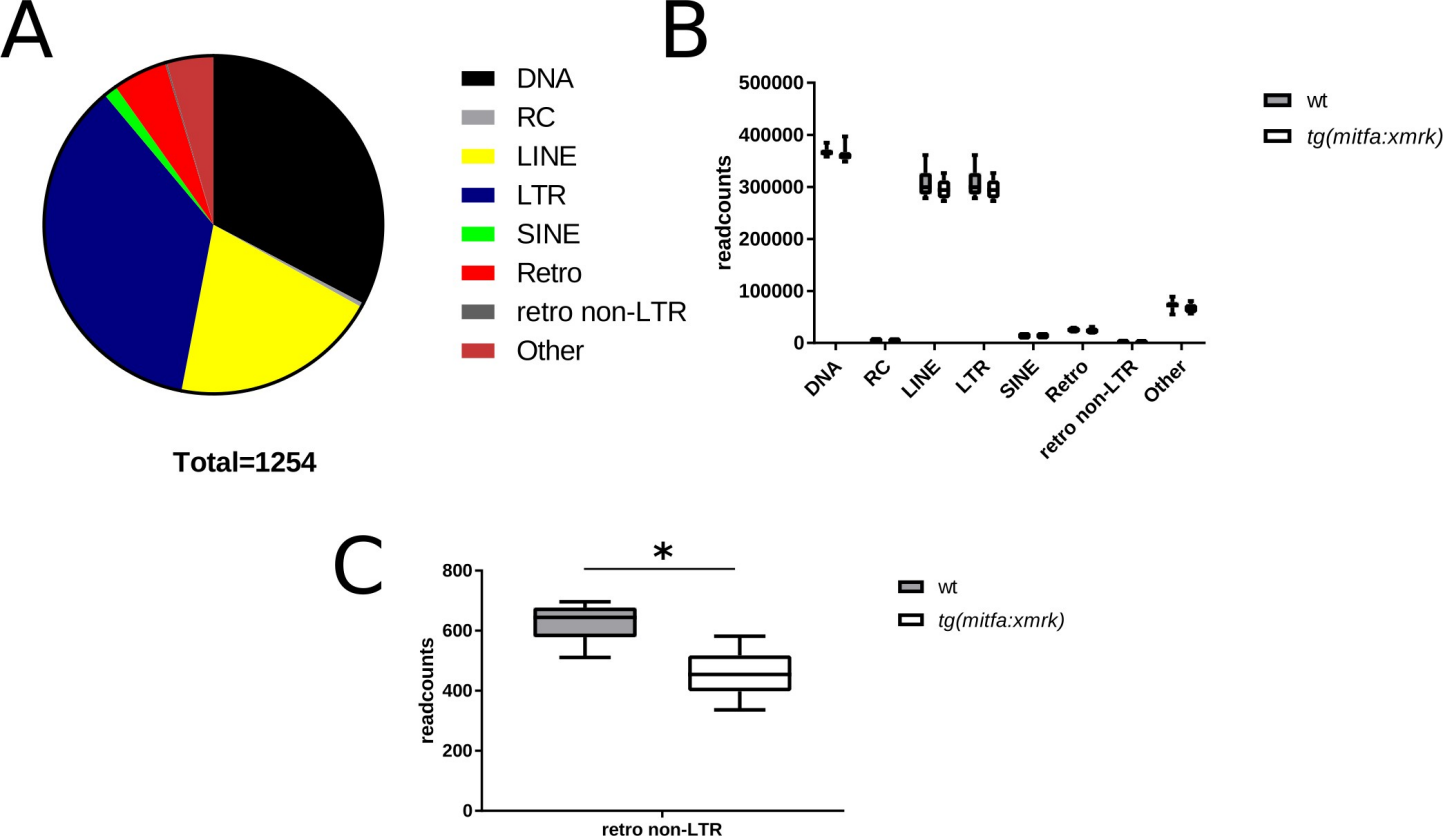
Families of expressed TEs found in RNA-seq data of wildtype and tg(mitfa:xmrk) larvae. (A) In total 1254 TE families were found to be expressed. A TE was considered as expressed, if at least one read was mapped to the consensus sequence. The diagram represents the percentage of the indicated TE categories in the total amount. (B) Sum of all DESeq normalized read counts of the indicated category in wt and transgenic fish for analyzing if one category is overexpressed in one of the samples. No significant differences in expression except for the retro non-LTR (C) could be observed. These elements have significantly more (* p<0.05) read counts in wildtype larvae.

**Fig 2:**
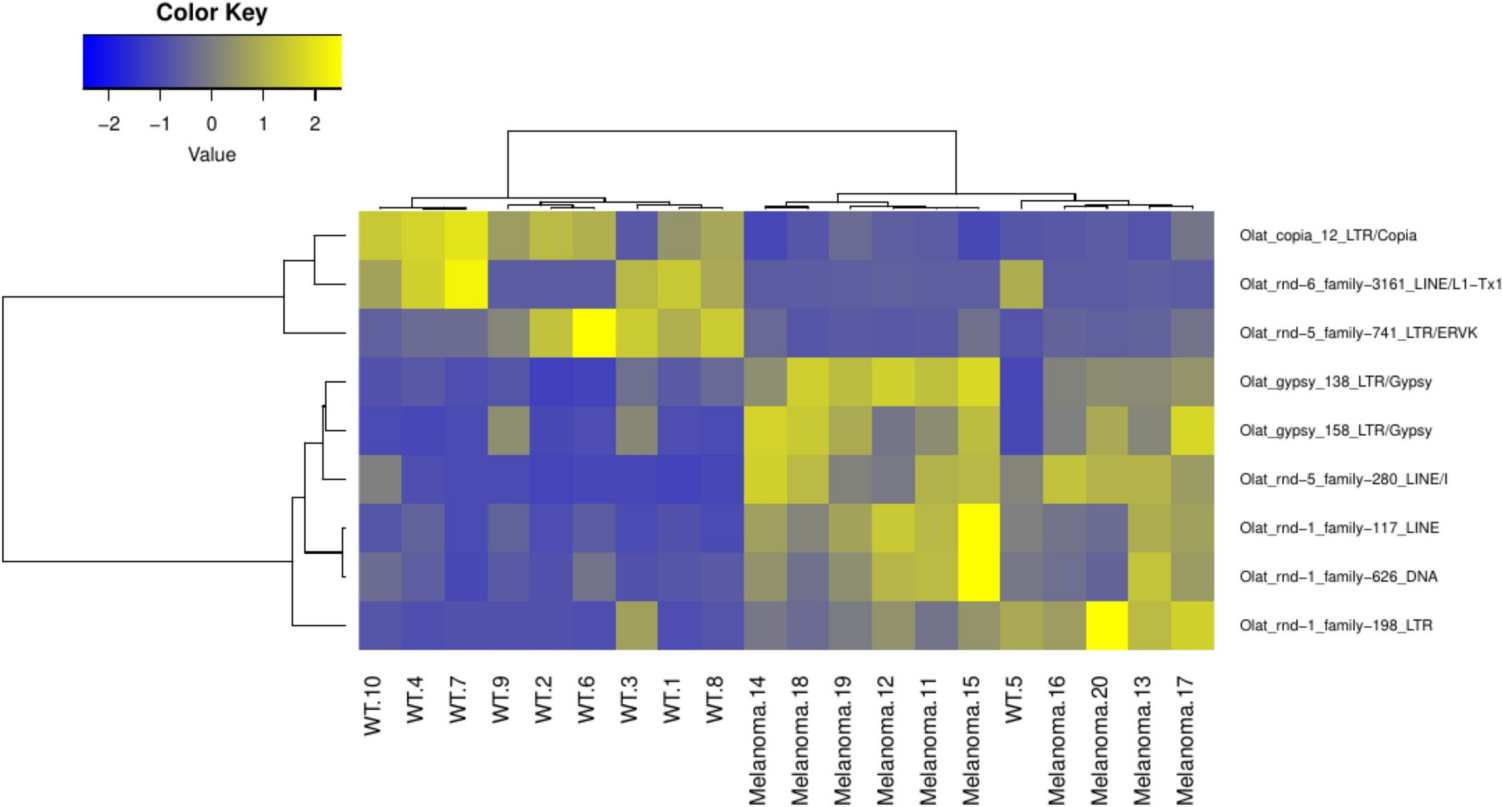
Heatmap of differentially expressed TE families. The analyses of differentially expressed TE families revealed distinct expression patterns of TEs. In wildtype (dataset 1–10) fish 3 TE families are overexpressed, whereas in melanoma bearing transgenic fish (dataset 11–20) 6 TE families are over-represented.

After mapping the RNA-seq data to the medaka specific TE library, we analyzed the total read counts of transcribed TEs in wt and *tg(mitfa*:*xmrk)* fish. For the selected TEs, we further checked if our mapped reads have hits in annotated genes. It reveals that ~25% (21201) of the reads have a hit in an exon (S1 Fig), corresponding to 111 genes. Of these, 13 genes are mono-exonic and are annotated as ‚novel transcripts’. All of these transcripts have TE specific domains and might de facto correspond to TE genes. In 75 genes the reads match in the last exon, in 23 genes in some other exon. For these genes we could not detect a significant expression change (log2FC: mean = -0.03, SD = 0.29). We found no correlation of mean expression level and log fold change between genes and TEs falling within these genes (0.04 and 0.02, respectively). We found in total 1254 expressed TE families (Fig 1A), where a TE was considered as expressed, if at least one read was mapped on it. Among them are LINEs (19,9%), SINEs (1,3%) and unclassified retroelements (5%). With 35,9% the LTR TEs are the largest group of expressed mobile elements. The amount of expressed non-LTR retroelements is only 0,15%. Surprisingly, we also found a high amount of DNA TEs (32,7%) and rolling-circle (RC) TEs (0,4%) in our datasets. In addition, expressed families comprise 4,5% of unclassified TEs, which could not be further characterized. By comparing the total normalized read counts of the expressed groups of TEs, we found no difference between wildtype and medaka tumor (Fig 1B) fish except for the non-LTR retroelements (Fig 1C). In this group there was a significant difference (p<0.05), with a higher expression in wt than in melanoma fish. After the global characterization of expressed TE elements, we analyzed our datasets for individual differentially expressed TE families (| logFC | > 1, pValue < 0.05 and DESeq2 basemean > 100) in wildtype and transgenic medaka. We found six upregulated families in transgenic melanoma fish (Fig 2). Among these families, there are two LINE (Olat_rnd-5_family-280#LINE/I and Olat_rnd-1_family-117#LINE), three LTR (Olat_rnd-1_family-198#LTR, Olat_gypsy_158#LTR/gypsy and Olat_gypsy_138#LTR/gypsy) and one DNA (Olat_rnd-1_family-626#DNA) elements. Three elements, two LTR (Olat_copia_12#LTR/Copia, Olat_rnd-5_family-741#LTR/ERVK) and one LINE (Olat_rnd6-_family-3161#LINE/L1) elements, are consistently downregulated in transgenic fish. We further investigated in detail, the read-distribution of these TEs, to see if they overlap with genes (S1 Table) found differentially expressed in melanoma fish in previous studies [41, 42]. We found that Olat_gypsy_158-LTR/Gypsy maps to ENSORLG00000024220 and ENSORLG00000027568, and Olat_rnd-5_family-280#LINE to ENSORLG00000027174. However, all three genes are annotated as ‚novel gene‘ and contain domains typical for transposons. For Olat_rnd-1_family-198#LTR 4.81% of all reads map to ENSORLG00000018143 (gene name: kidins220b), however log2FC for this gene is only 0.02.

### Characterization of differentially expressed TE families

Several medaka TEs have been previously characterized including the well-known Tol-2 element, as well as the Swimmer-1 and gamera-like TE elements [35, 54].

**Fig 3:**
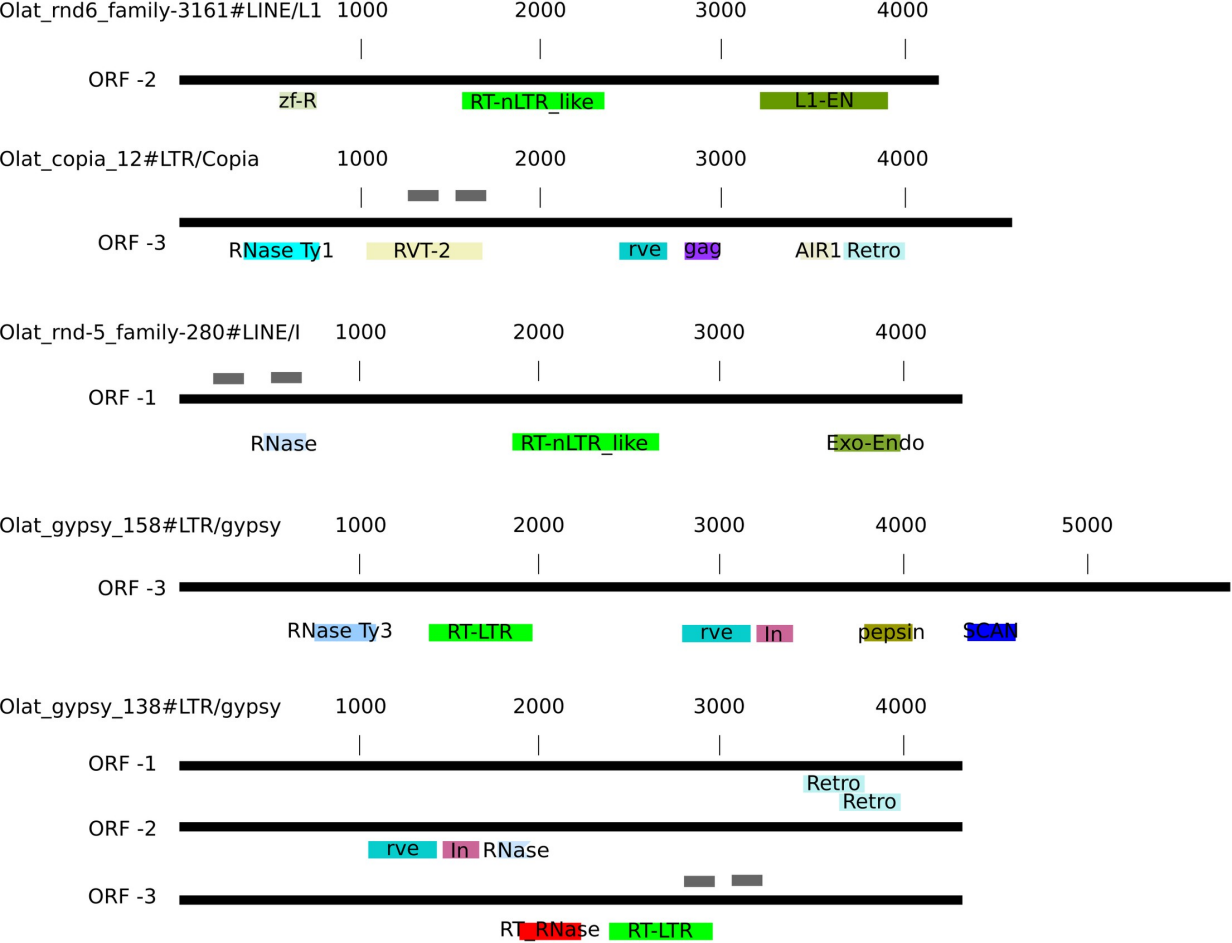
Schematic ORF distribution of the differentially expressed TE families. The predicted ORFs with characteristic TE domains are indicated. The numbers represent the length (in base pairs) of the TEs. The different domains are highlighted in different colors. The primers used for qRT-PCR analysis are indicated in gray, above the corresponding TE. For Olat_rnd−5_family−280#LINE/I, Olat_gypsy_138#LTR/gypsy, and Olat_copia_12#LTR/Copia the domain structure of the genomic locus indicated in the results part is shown; they are encoded on the reverse strand.

**Fig 4:**
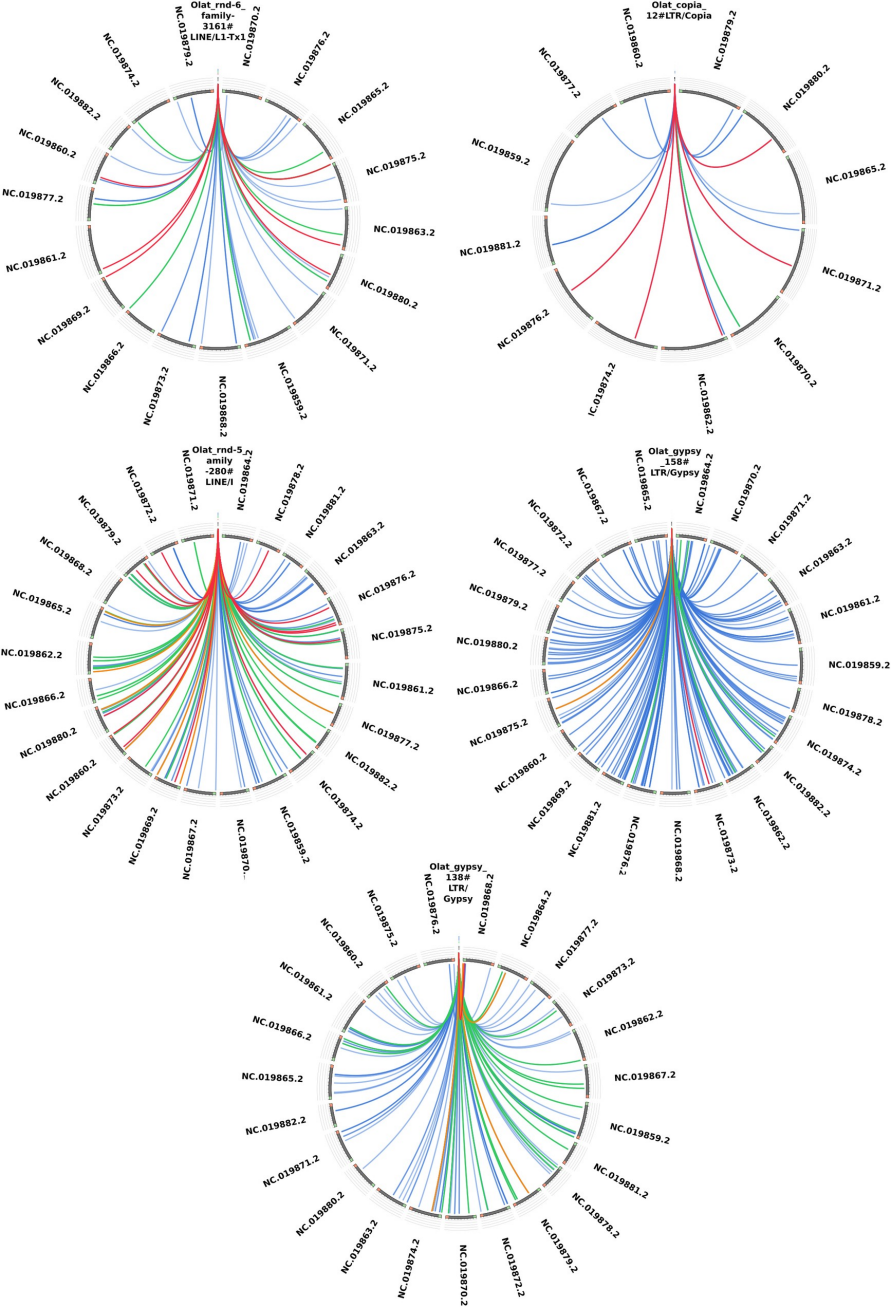
Visualization of the blast hits. The blast hits of the individual TE sequences are shown. The score of each blast hit was divided by the maximum reached within the blast search (score/max bitscore). The colour coding represents the ratio of the score with blue< = 0.25, green< = 0.70, orange< = 0.90, and red>0.90. A score of >0.90 was assumed as a full-length hit within the genome.

For further characterization, we analyzed the domain structure of the TE families that were found to be differentially expressed (Fig 3) with the NCBI domain structure tool [50, 51]. For Olat_rnd-5_family-741#LTR/ERVK, Olat_rnd-1_family-198#LTR, Olat_rnd-1_family-626#DNA and Olat_rnd-1_family-117#LINE no characteristic TE domains could be predicted with NCBI domain finder and we excluded them from further analysis. For the other TEs, several specific TE domains were found within the consensus sequence. A blast search [44] (Fig 4) revealed several hits in the medaka genome. In addition, no similarities with previously published medaka TE sequences could be observed. We also used the consensus2genome tool to identify full length TEs within the genome (S2 Fig). Additionally, this tool shows the frequency at which each part of the TE is found in the genome. For all LTR elements, except for Olat_gypsy_138#LTR/Gypsy, 3’ and 5’ LTRs are frequently more found. For the differentially expressed LINEs and for Olat_gypsy_138#LTR/Gypsy, the consensus sequences are more homogeneously represented within the medaka genome.

In detail, the search for our TE sequences in the medaka genome resulted in six full length hits for Olat_rnd-6_family-3161#LINE/L1-Tx1 and five for Olat_copia_12#LTR/Copia, which both are downregulated in tumors. For the TEs upregulated in tumors, 13 full length sequences (by blast search) and 12 full length sequences (with the consensus2genome tool) for Olat_rnd-5_family-280#LINE/I were found. For Olat_gypsy_158#LTR/Gypsy and Olat_gypsy_138#LTR/Gypsy one full length copy each were found within the medaka genome. The full-length sequences found are marked up in red in Fig 4. A lot of other hits could be observed for these TEs within the medaka genome. Most of these hits resemble partial sequences of the TE sequences hinting to jumping events in the past.

We then investigated the domain structure of these full length hits to characterize, if these sequences may be full length transposons and inferred the domain structure of the most promising ones (Fig 3).

The six full length copies of Olat_rnd-6_family-3161#LINE/L1 contain mostly two ORFs with two or even three predicted distinct open reading frames (ORFs), although sometimes the domains are disrupted. At chr3:2856757–2860916 within the medaka genome we found a potential complete copy of this TE with one ORF in which the typical LINE TE structures can be found (Fig 3). This ORF encodes a LINE-1 endonuclease domain (L1-EN, Accession number cd09076), a RT-like domain of non LTR-retroviruses (accession number cl01650) and a zinc-binding domain of reverse transcriptases (accession number cl016506).

The downregulated copia element (Olat_copia_12#LTR/Copia) has five potential full length hits within the genome. At position chr13:19523091–19527882 the sequence is clearly flanked by two LTR sequences and one ORF encoding an RNase HI (accession number cd09272) domain, which is typical for the TY1/copia family [40], can be found. In addition, a reverse transcriptase domain of the RVT_2_super family (accession number cl06662), an integrase core domain (rve, accession number pfam00665) and a gag-domain (accession number pfam13976) were predicted within this sequence, as well as a partial AIR1 super family domain (accession number cl34894) and two partial gag domains (accession number cl26047 and pfam 13976). For Olat_rnd-5_family-280#LINE/I we found at chr16:7446391–7450254 a sequence with one ORF containing three characteristic LINE TE domains followed by a polyA signal. Within the ORF, an endonuclease-reverse transcriptase (accession cl00490) and a RT-like domain of non-LTR retrotransposons (accession number cd01650), along with a partial RNaseHI domain (accession cd09276) were predicted. Furthermore a RNaseH domain was predicted within the sequence of Olat_rnd-5_family-280#LINE/I.

Olat_gypsy_158#LTR/gypsy is composed of one ORF, encoding different proteins with a reverse transcriptase (accession number cd01647), an RNase (accession number cd09274) and two integrase domains (pfam00665 and pfam17921). In this ORF are also predicted a retropepsin-like protein (cd00303) and a SCAN domain (accession number cd07936).

The single potential full length copy of Olat_gypsy_138#LTR/gypsy at chr10:1520943–1525204 has three different ORFs and is flanked by typical LTR sequences. One ORF encodes a protein with a retrotransposon gag domain (cl29647). The second ORF encodes integrase domains (accession numbers pfam00665 and pfam17921), a CHROMO domain (accession number cl28914) and partial pepsin-like aspartate proteases (accession number cl11403). In addition to these ORFs, a partial RNase domain (accession number cl14782) was found. The third ORF encodes the reverse transcriptase domain (accession number cd01647) and a second more complete RNaseH-like domain (pfam17919) which can be found in reverse transcriptases.

### TE sequences are expressed in full length

As known from previously described medaka TEs, like the Rex elements [55–58] some of them are not expressed in full length. We investigated the read distribution of differentially expressed TEs in wildtype and transgenic fish by mapping the RNA-seq reads to the consensus sequences from our TE library. The derived histograms show the corresponding read distribution over the whole TE sequence (S3 Fig). Most of the differentially expressed TEs have hits over the entire sequence. The density of the read-counts corresponds well with the differential expression of the TEs. For Olat_rnd−1_family−198#LTR most of the reads are mapping within the first 120 bp. For Olat_rnd−5_family−280#LINE/I the distribution of the readcounts covers the entire sequence, but more reads are mapped to the last 1000 bp, where the partially RNase domain is predicted in the consensus sequence.

### Validation of differentially expressed TE families by qRT-PCR

**Fig 5:**
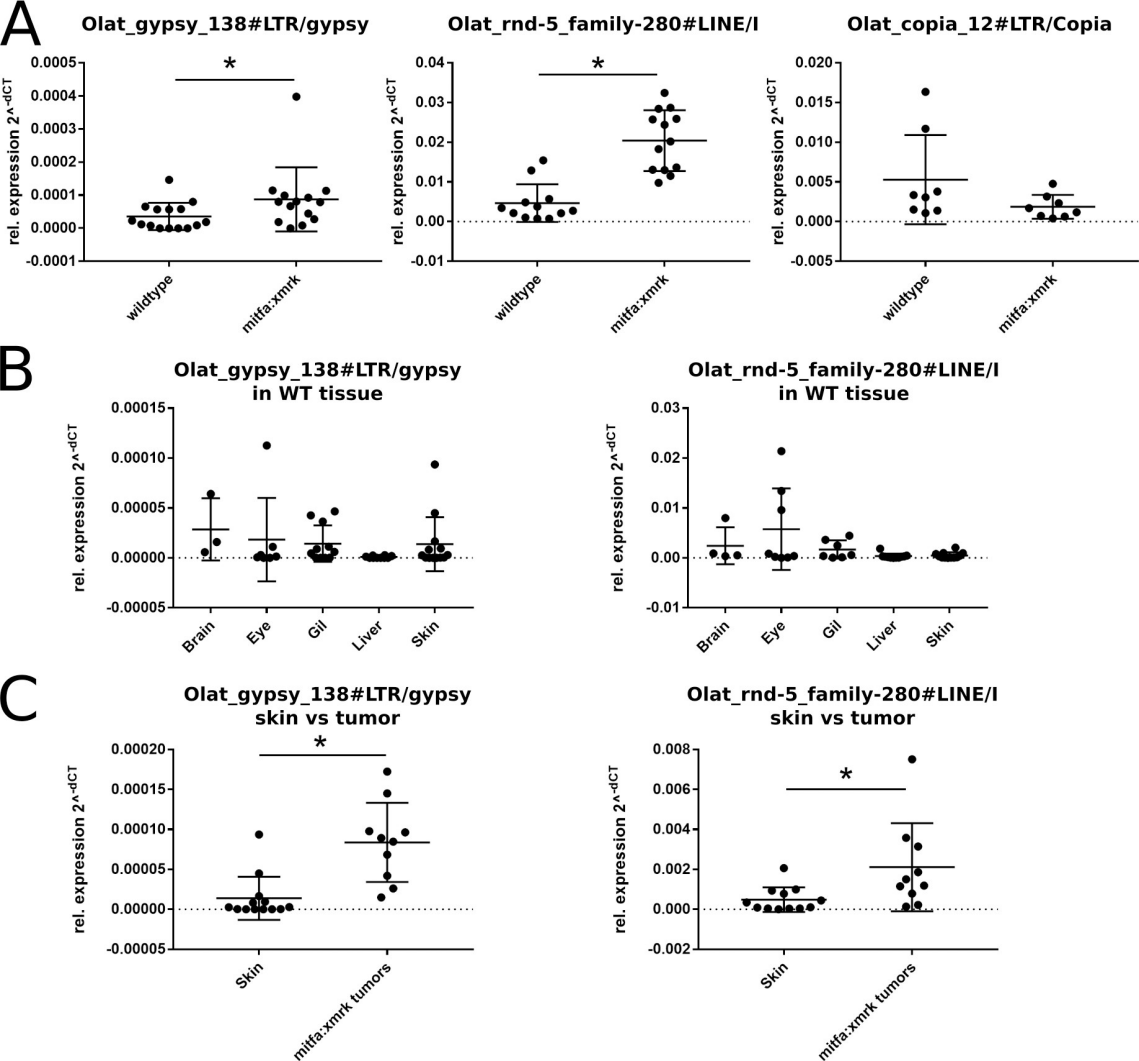
Expression of TEs in larvae and adult tissues. (A) Expression of three TE families in wildtype and transgenic larvae. The higher expression of LTR gypsy 138#LTR and Olat_rnd5_familiy-280_LINE/1 in melanoma developing larvae was confirmed by qRT-PCR. (B) Expression of TE familes in wildtype tissues. (C) Expression of both TE families in skin and melanoma tumors.

Next, we evaluated the results from the RNA-seq data by qRT-PCR. For further analyses, we chose TEs in which a TE-specific domain structure was predicted (Fig 3) and which we considered as being completely expressed. Primers were designed for three upregulated TE families, Olat_gypsy_138#LTR/gypsy (logFC = 111.07, pVal<0.05, baseMean = 111.07), Olat_gypsy_158#LTR/gypsy (logFC = 1.70, pVal<0.05, baseMean = 1852.48), Olat_rnd5_family-280#LINE/I (logFC = 1,53, pVal<0,05, baseMean = 1233.42) and one downregulated TE family, Olat_copia_12#LTR (logFC = -1.05, pVal<0.05, basemean = 122.41) in melanoma fish. The significantly higher expression of Olat_gypsy_138#LTR/gypsy and Olat_rnd5_family-280#LINE/I could be validated by qPCR (Fig 5A). In contrast, a statistically significant downregulation of the Olat_copia_12#LTR element and upregulation of Olat_gypsy_158_LTR/gypsy could not be shown. For these TEs only a low tendency toward the predicted expression status was observed by qRT-PCR.

### Expression of Olat_gypsy_138#LTR/gypsy and Olat_rnd5_family-280#LINE/I in adult tissue

After having confirmed the expression of candidate families on juvenile medaka larvae from which the RNA-seq data were generated, we analyzed the expression of these two TE families in non-tumorous tissues in comparison with malignant melanoma of adult *tg(mitfa*:*xmrk)* fish. qRT-PCR was performed on brain, eye, gills, liver and skin of wildtype medaka (Fig 5B). The expression of Olat_gypsy_138#LTR/gypsy varies between wildtype organs with the lowest expression in liver. A similar expression pattern was observed for the Olat_rnd5_family-280#LINE/I. By comparing the expression of these TE families in wildtype skin with melanoma from the transgenic line (Fig 5C), a significant difference in the expression could be detected, indicating a melanoma specific upregulation of both TEs also in adult fish, with more advanced tumors than in the juvenile individuals.

## Discussion

In this study we compared expression of TE families by RNA-seq in juvenile wildtype and transgenic medaka carrying the *xmrk* oncogene under the control of the *mitfa* promoter. The transgenic fish develop malignant melanomas, which are on the histological and molecular level very similar to the tumors found in humans [38, 39]. In this study the TE expression was analyzed with a self-developed medaka-specific TE library. We used a similar approach as previously proposed by others to build TE libraries [33] to detect the consensus sequences of TEs within the medaka genome. In addition, we used tools dedicated to the search of certain TE families, which indeed uncovered additional transposons. With our approach, we identified the consensus sequences of over 4000 different transposon families in the medaka genome, compared to the previous 1364 entries in FishTEDB for this model organism [33]. The masking of the genome with our library led to a coverage of 34.5% of the genome (33.6% without simple repeats), which is slightly higher than the coverage previously obtained by Chalopin et al [22] (28% without simple repeats). This indicates that we have a more complete library now. It is true, however, that some redundancies might remain in this library, for instance due to artifactual consensus formed by nested insertions. The largest fraction of TE families are DNA and LINE transposons, whereas the number of SINE families is very low. As in other studies we found several unknown elements, which cannot be assigned to any known TE group. In comparison to zebrafish, where over 50% of the genome is TE-derived [25], we identified for the medaka genome only 34% of TE-derived sequences. This is in the range of the transposon content reported in other studies for this model organism [22, 33].

In human cancers a LINE1 element activation was found, which can lead to new genomic insertions [59] and genomic instability. In addition, TE activity was observed in human melanoma and melanoma-derived cell lines [60]. Such findings motivate studies to investigate if TEs are more active, or if particular TE families become activated, in malignant melanoma.

Using an RNA-seq approach, we identified 1254 expressed transposon families in wildtype and tumor bearing fish. All major TE families were represented in the RNA-seq data. With our approach, by using a polyA enriched RNA library, we are aware, that we might miss expressed TEs, which do not require a polyA-tailed intermediate [30]. This might be circumvented by using a total RNA library, but might have the drawback, of enhancing the background noise, by adding more intronic non-complete TE sequences [61]. Surprisingly, a large amount of DNA transposons were detectable in our data, which are not expected in polyA enriched cDNA. However, as recently reviewed, there are indications, that these TEs can indeed be polyadenylated [61]. Another explanation for our result is the fact that some protein coding genes are derived from TEs during evolution and thus by homology identification appear in our RNA-seq datasets [62].

The five sequences, in which characteristic TE domains could be found, have very few full length hits in the medaka genome. With 12 full length sequences the most frequently found TE was the Olat_rnd-5_family-280#LINE/I. We also observed a lot of Olat_rnd-5_family-280#LINE/I sequence fragments within the genome, hinting towards an evolutionary young TE acquired by medaka. The significantly upregulated Olat_gypsy_138#LTR/Gypsy has also a lot of hits within the genome, but only one full length sequence could be found.

The differential expression analysis revealed several upregulated TE families specifically in the melanoma developing transgenic fish. The expression is specific to the melanoma tumors and these TEs show only sporadicly spontaneous upregulation in adult wildtype tissue. The lowest expression of the analyzed LINE and LTR elements was found in liver. Interestingly, the LINE element appears to be transcribed in wildtype larvae too but it is more highly expressed in a tumorous state. The LTR element upregulated in tumorous fish has a very low expression in wildtype. Expression of the two TEs studied in more detail was significantly increased in melanomas of adult fish compared to healthy skin of wildtype fish. This upregulation is intriguing because increased TE activity can lead to further mutations, rearrangements and instability within the genome [3]. In particular LINE elements can induce DNA damage and double strand breaks [63, 64].

An increased TE activity in melanoma is in line with observations of other human derived tumors, where increased TE activity was previously described [11]. Other studies associated increased TE activity with an increased metastatic potential as a result of demethylation of the tumor cell genome [10]. Tumors like melanoma often show an altered methylation pattern of the genome. DNA methylation suppresses TE activity. Consequently, an altered methylation pattern can increase the expression of previously silenced TE [13].

While it is unclear if the TEs we identified are still able to jump within the genome, due to accumulated mutations or stop codons within their sequence, it is very interesting that an increase in TE expression is observed in melanoma. Even if only some parts of the TEs are active, these parts and domains contribute to genomic instability by interacting with each other and enabling smaller non-autonomous SINE or other sequences to propagate [65]. These sequences would not have been detectable with our approach, but can be investigated in future studies.

In summary, our study shows that some TE families present an increased activity in malignant melanomas compared to non-tumorous tissues. As TEs can lead to genomic rearrangements, gene disruptions or alter gene expression level, they should be considered as a source of genomic instability.

## Supporting information

S1 Fig Read distribution of mapped sequences within the medaka genome. The majority of the reads map within intergenic regions and introns of the genome. Only 25% of the reads have a hit in an exon.
(TIF)

S2 Fig TE consensus analyses within the medaka genome. The consensus sequences of the differentially-expressed TEs, which show clear TE protein domain similarities, were used as input for the consensus2genome tool. The divergence to the consensus sequence of the corresponding TE found in the genome are shown (left). In red are highlighted full length sequences, ie genomic hits covering at least 90% of the TE consensus sequence. In addition the coverage of the consensus is shown (right).
(PDF)

S3 Fig Read distribution within TE sequences in wt and transgenic medaka. The mapped reads are shown within the TE sequence. The heights of the bars are indicating the number of reads mapping to the corresponding part of the TE sequence.
(PDF)

S1 Table Read distribution of differential expressed TEs. (XLSX)

S1 File Medaka specific TE library. (FA)
