# Supplementary figures and images for: Differential expression of transposable elements in the medaka melanoma model
Source: PLoS One. 2021 Oct 27;16(10):e0251713. doi: 10.1371/journal.pone.0251713 (PMC8550402; doi:10.1371/journal.pone.0251713)

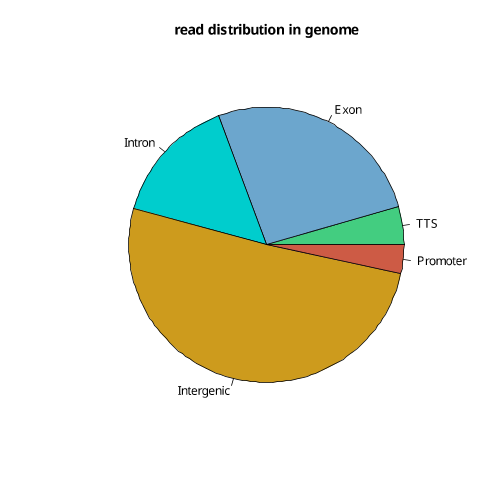

Supplement: S1 Fig — The majority of the reads map within intergenic regions and introns of the genome. Only 25% of the reads have a hit in an exon. (TIF) [file pone.0251713.s001.tif]
