# Supplementary material for: Differential expression of transposable elements in the medaka melanoma model
Source: PLoS One. 2021 Oct 27;16(10):e0251713. doi: 10.1371/journal.pone.0251713 (PMC8550402; doi:10.1371/journal.pone.0251713)
Supplement: S2 Fig — The consensus sequences of the differentially-expressed TEs, which show clear TE protein domain similarities, were used as input for the consensus2genome tool. The divergence to the consensus sequence of the corresponding TE found in the genome are shown (left). In red are highlighted full length sequences, ie genomic hits covering at least 90% of the TE consensus sequence. In addition the coverage of the consensus is shown (right). (PDF) [file pone.0251713.s002.pdf]

TE: Olat_rnd-6_family-3161-LINE/L1-Tx1
consensus size: 4171bp; fragments: 57; full length: 6 (>=90%)

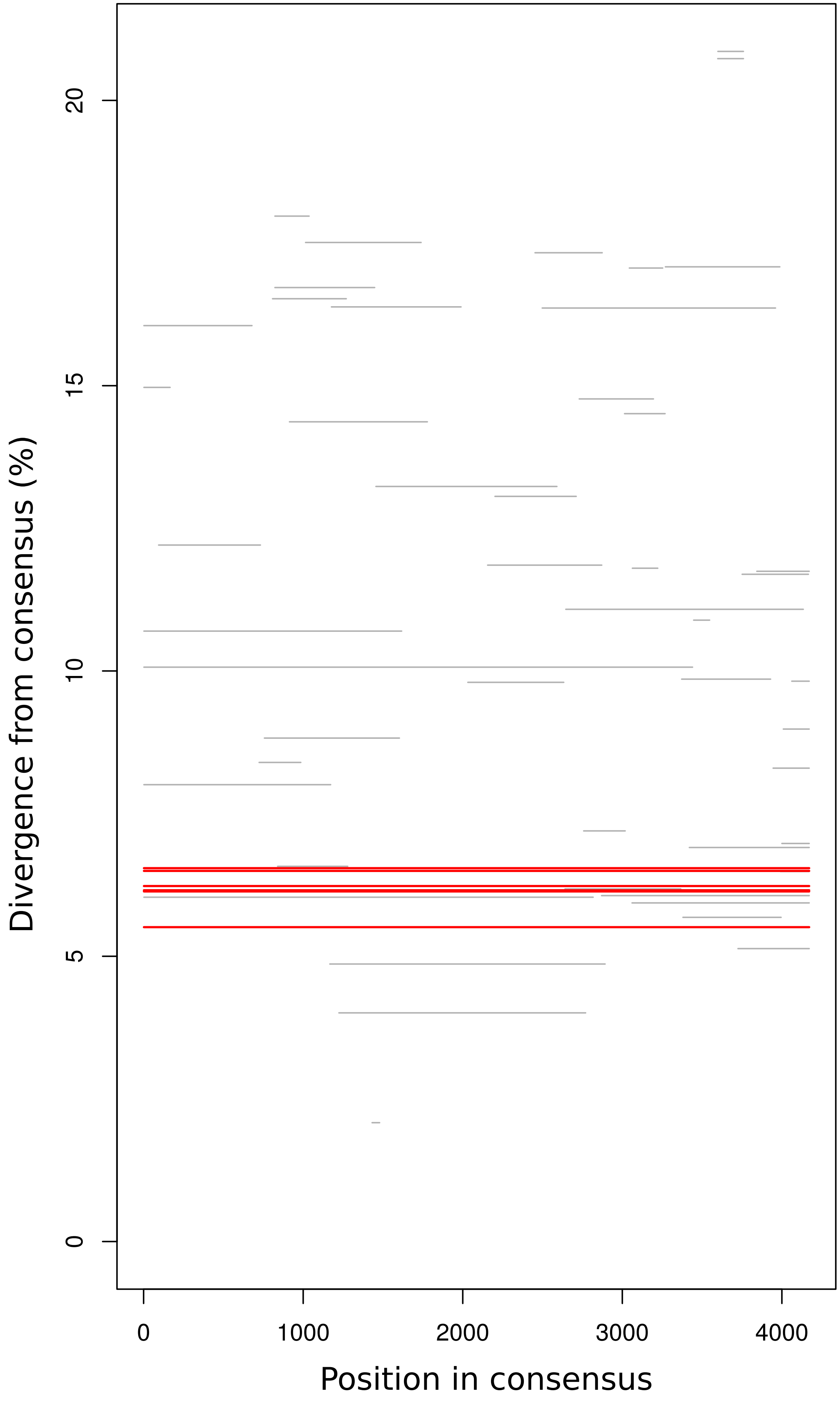

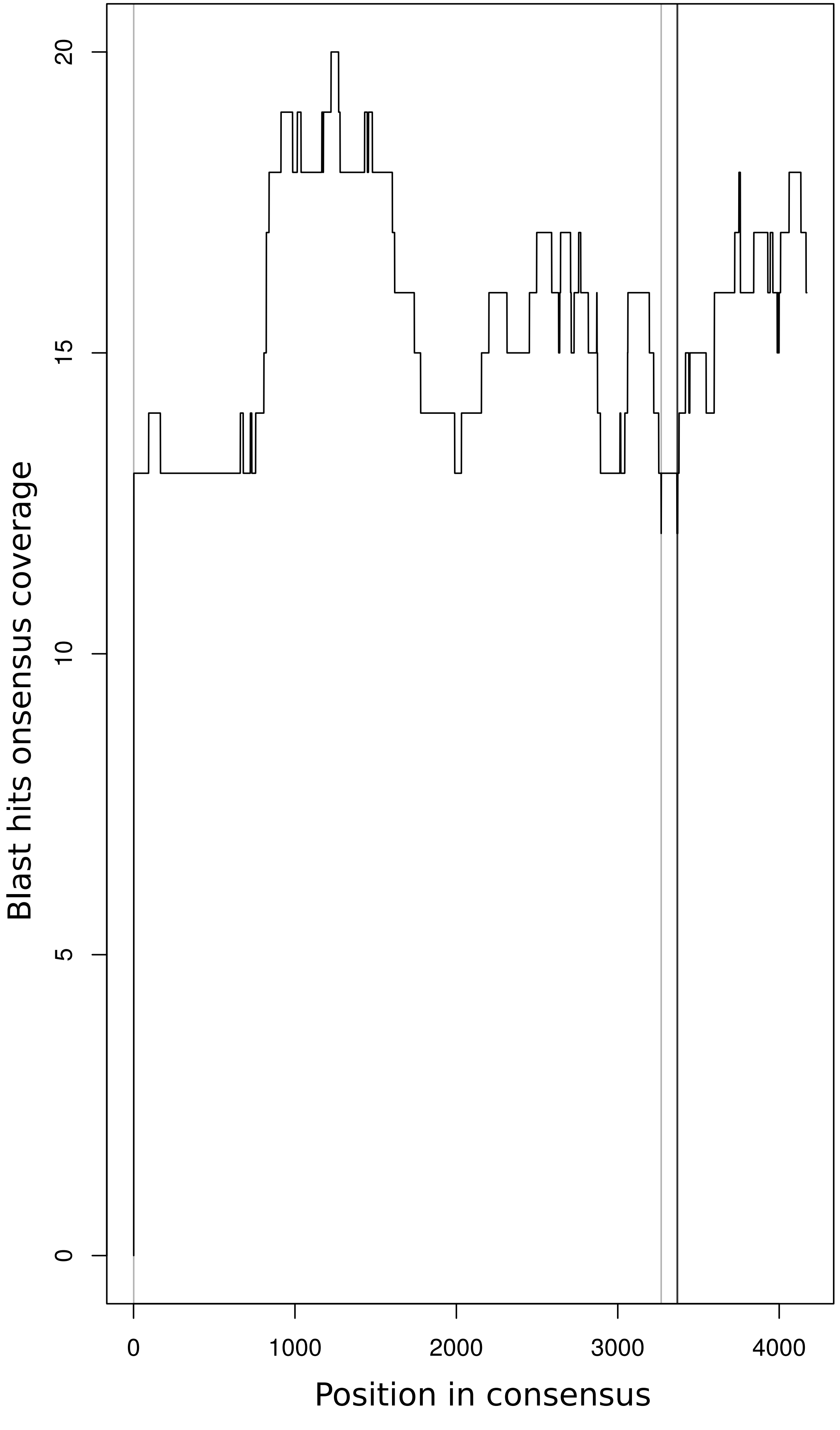

TE: Olat_copia_12-LTR/Copia
consensus size: 4796bp; fragments: 39; full length: 5 (>=90%)

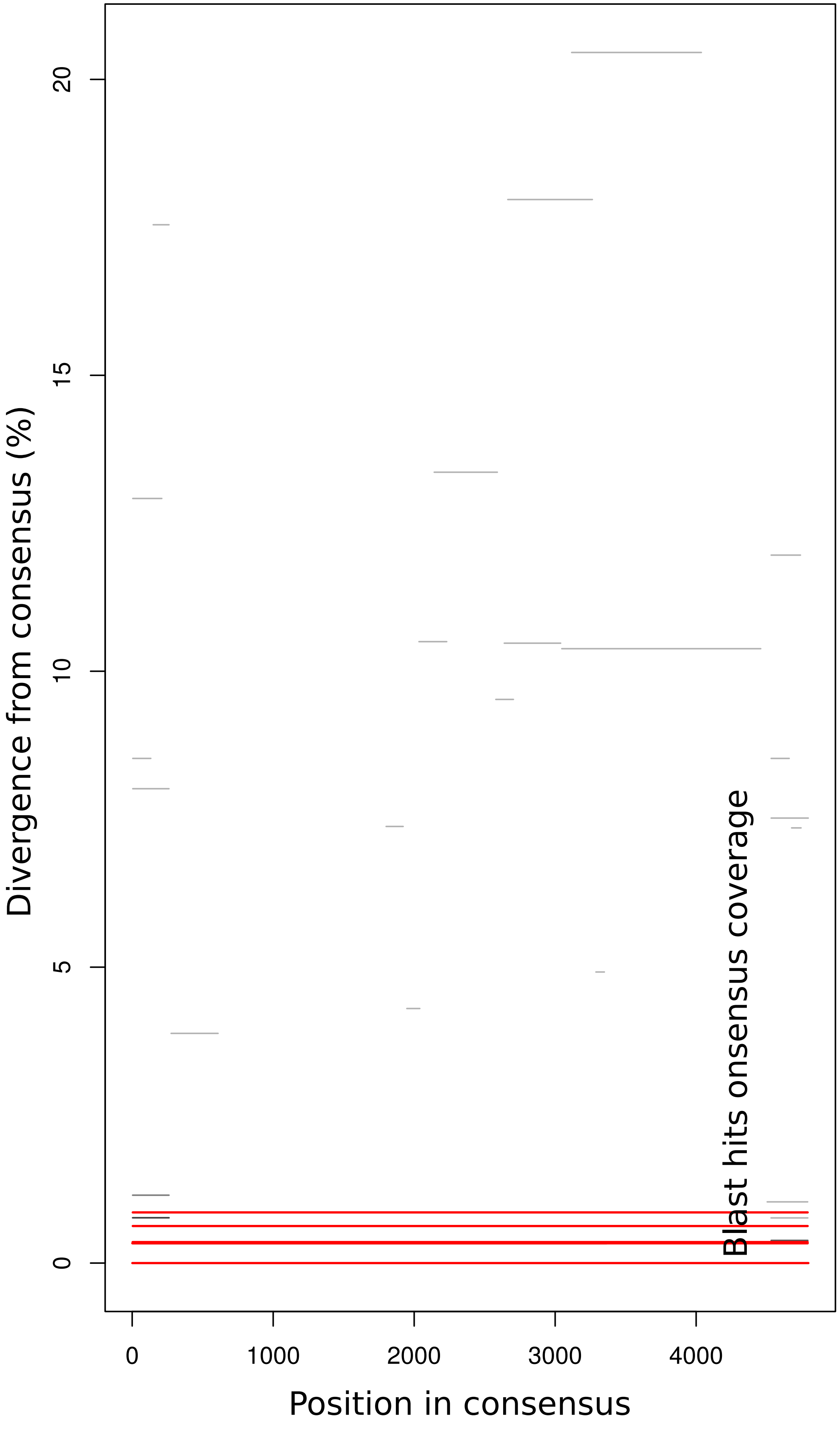

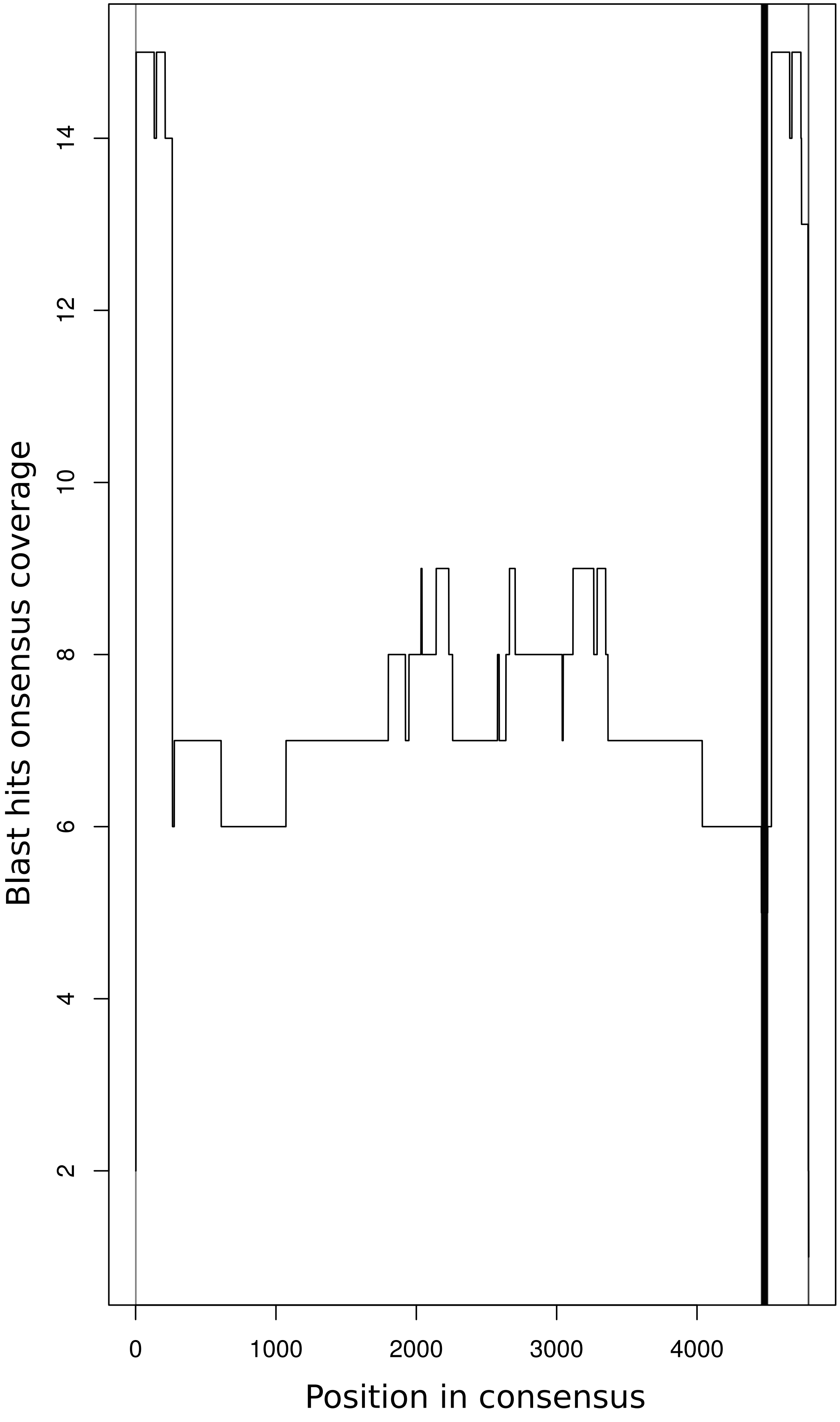

TE: Olat_rnd-5_family-280-LINE/I
consensus size: 4199bp; fragments: 205; full length: 12 (>=90%)

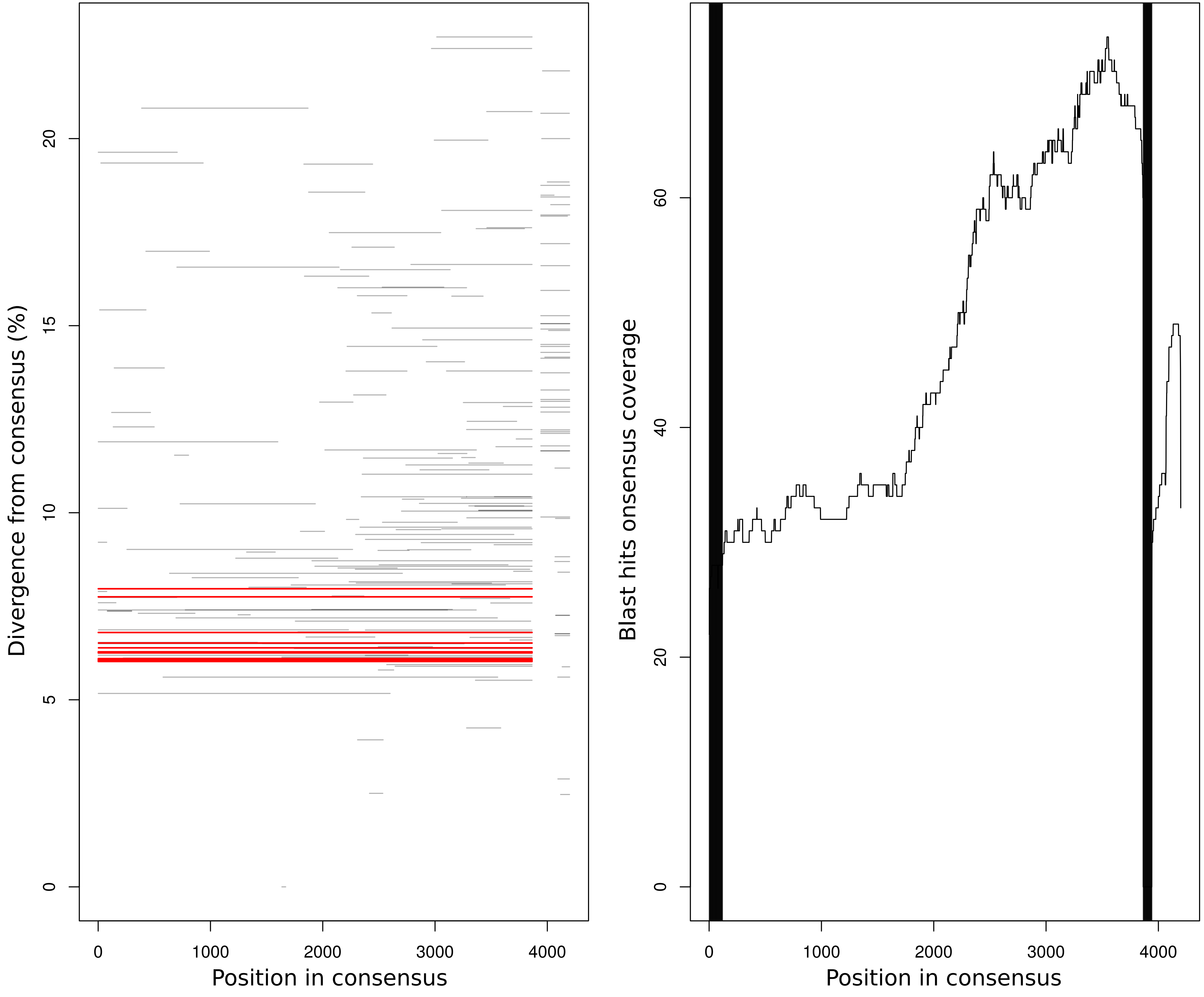

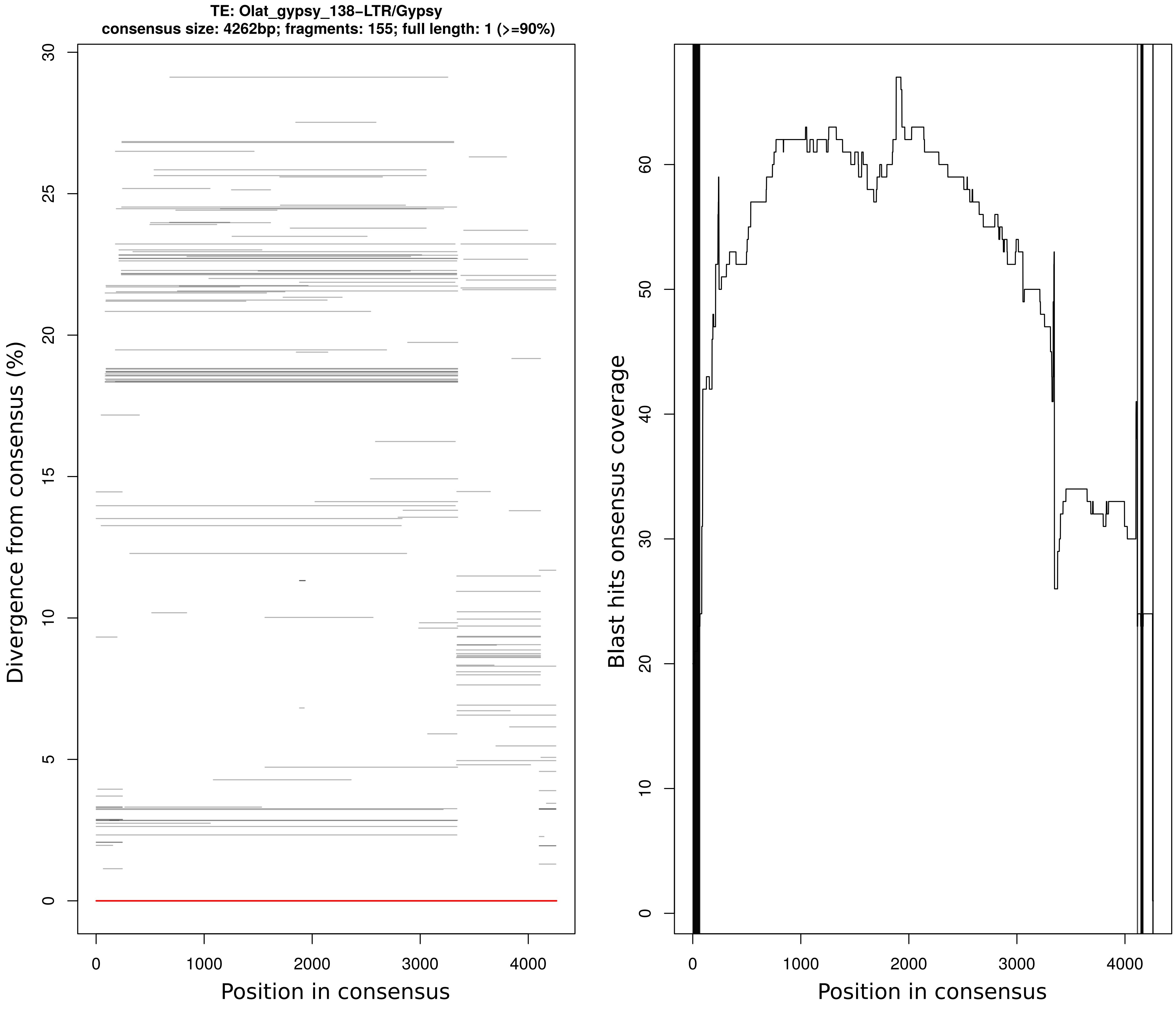

TE: Olat_gypsy_138-LTR/Gypsy
consensus size: 4262bp; fragments: 155; full length: 1 (>=90%)
Divergence from consensus (%)
0
5
10
15
20
25
30
Position in consensus
0
1000
2000
3000
4000
Blast hits onsensus coverage
0
10
20
30
40
50
60
Position in consensus
0
1000
2000
3000
4000

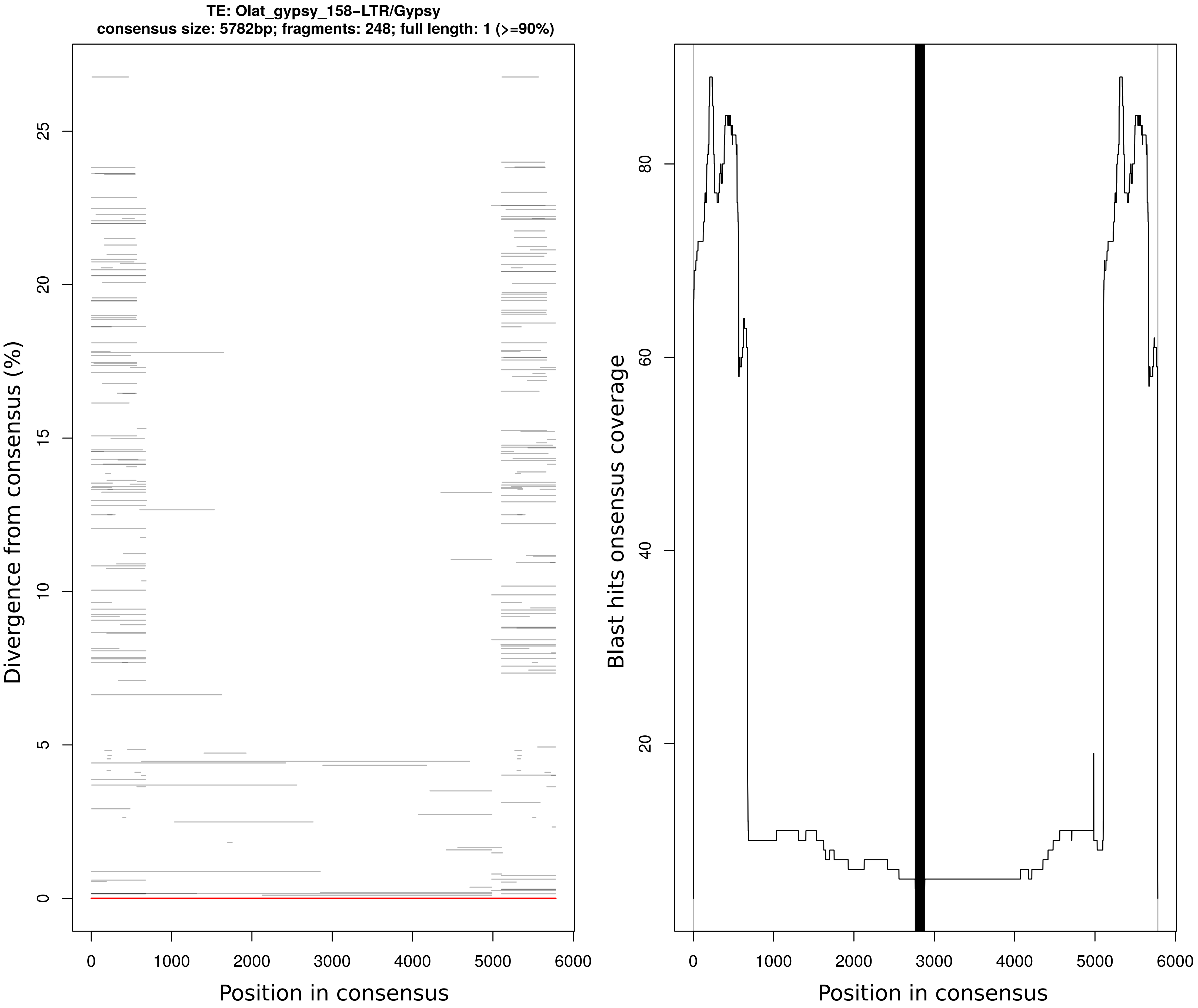
TE: Olat_gypsy_158-LTR/Gypsy
consensus size: 5782bp; fragments: 248; full length: 1 (>=90%)
Divergence from consensus (%)
Position in consensus
Blast hits onsensus coverage
Position in consensus
