# Supplementary material for: Differential expression of transposable elements in the medaka melanoma model
Source: PLoS One. 2021 Oct 27;16(10):e0251713. doi: 10.1371/journal.pone.0251713 (PMC8550402; doi:10.1371/journal.pone.0251713)
Supplement: S3 Fig — The mapped reads are shown within the TE sequence. The heights of the bars are indicating the number of reads mapping to the corresponding part of the TE sequence. (PDF) [file pone.0251713.s003.pdf]

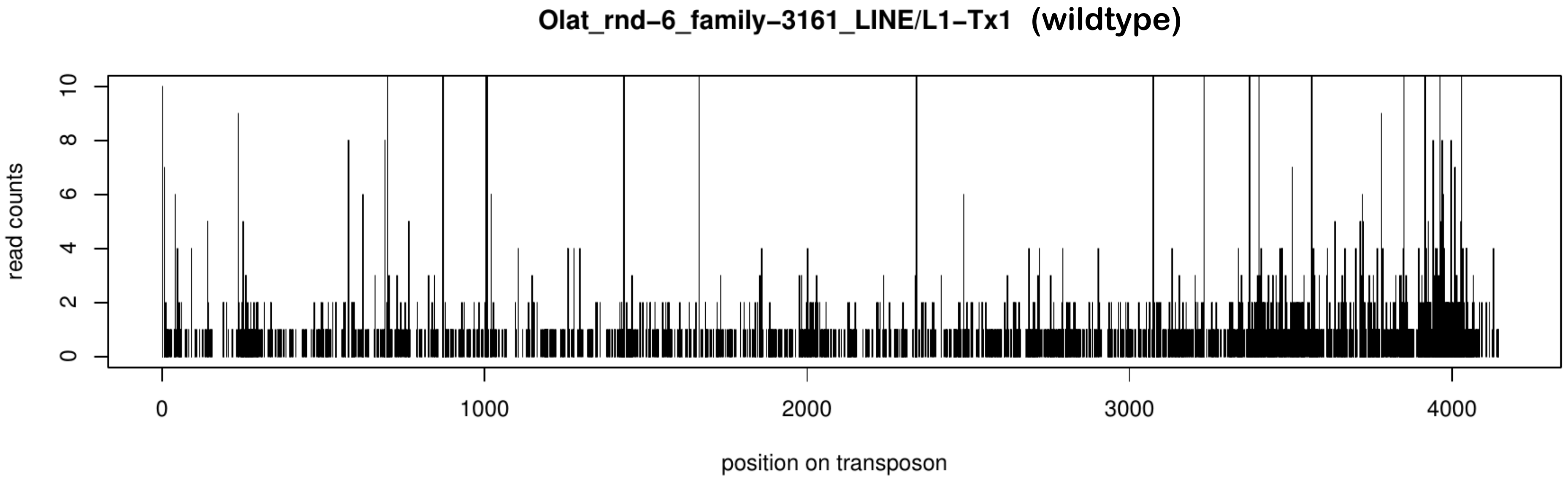

Olat_rnd-6_family-3161_LINE/L1-Tx1 (wildtype)
read counts
0
2
4
6
8
10
0
1000
2000
3000
4000
position on transposon

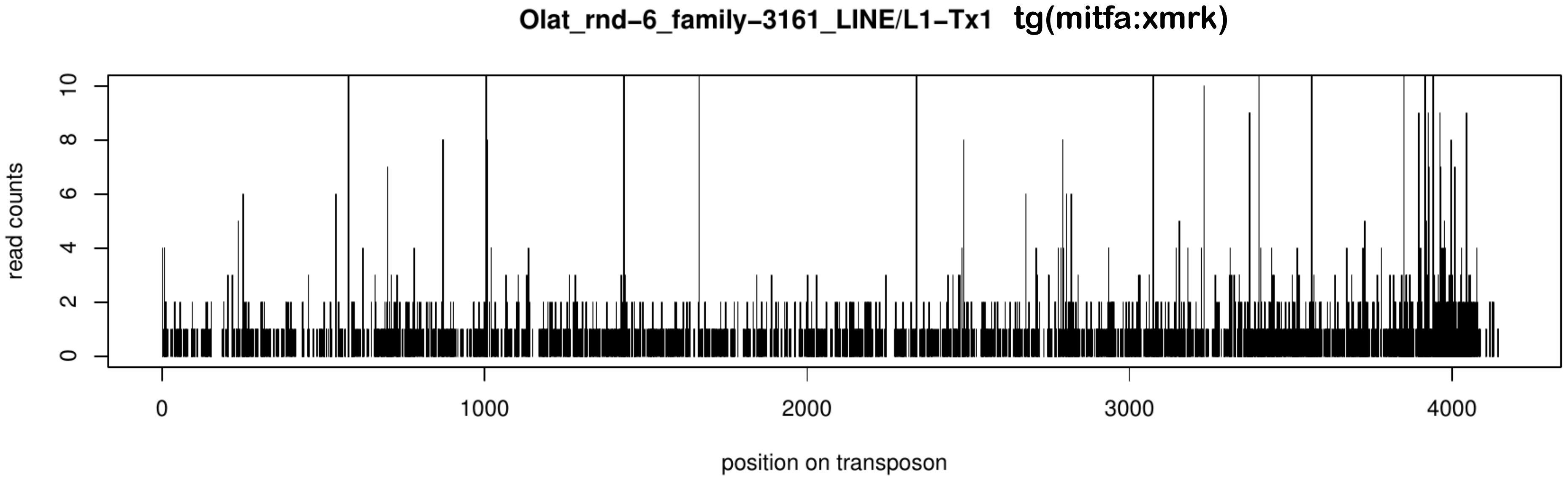

Olat_rnd-6_family-3161_LINE/L1-Tx1 tg(mitfa:xmrk)
read counts
0
2
4
6
8
10
0
1000
2000
3000
4000
position on transposon

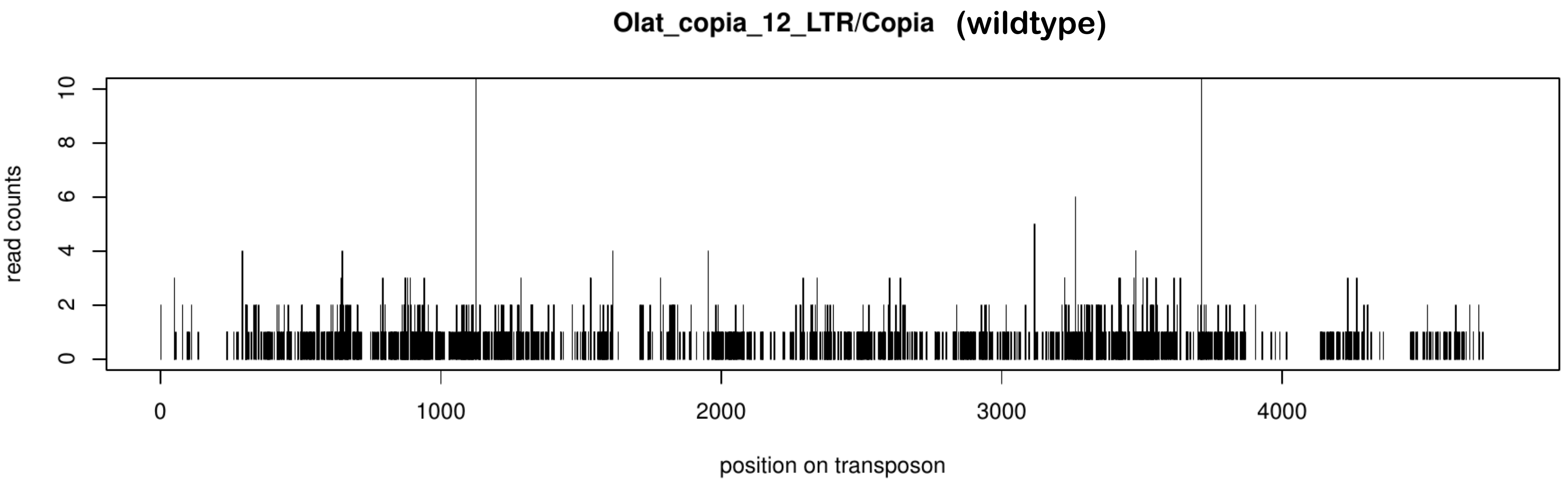

Olat_copia_12_LTR/Copia (wildtype)
read counts
0
2
4
6
8
10
0
1000
2000
3000
4000
position on transposon

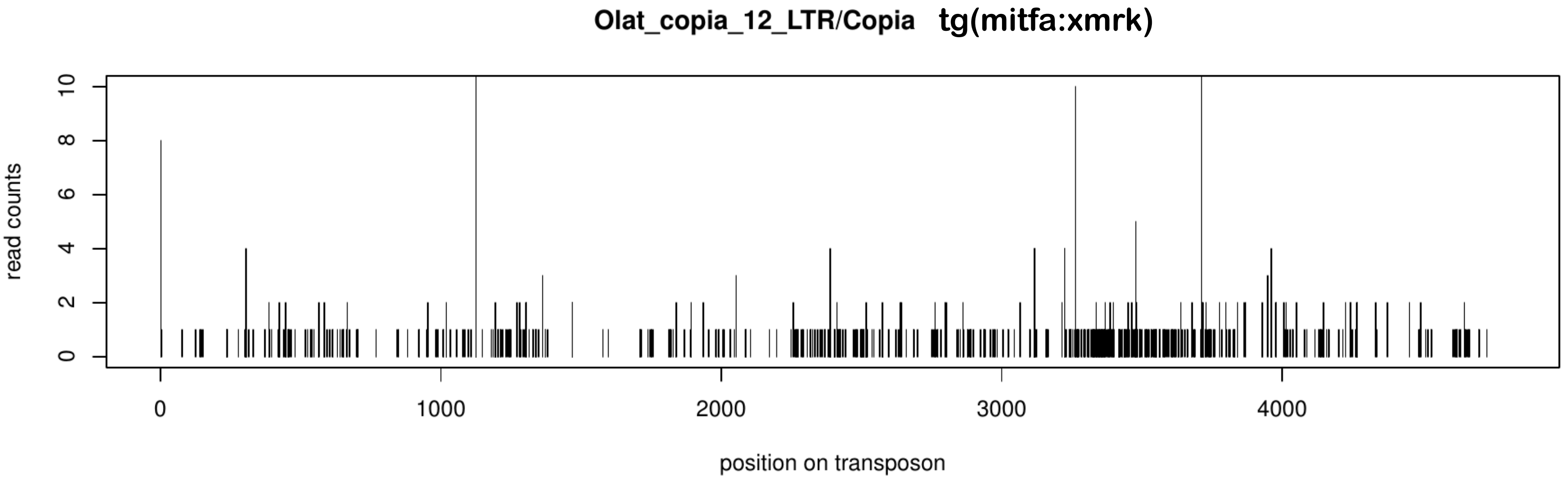

Olat_copia_12_LTR/Copia tg(mitfa:xmrk)
read counts
0
2
4
6
8
10
0
1000
2000
3000
4000
position on transposon

Olat_rnd-5_family-741_LTR/ERVK (wildtype)

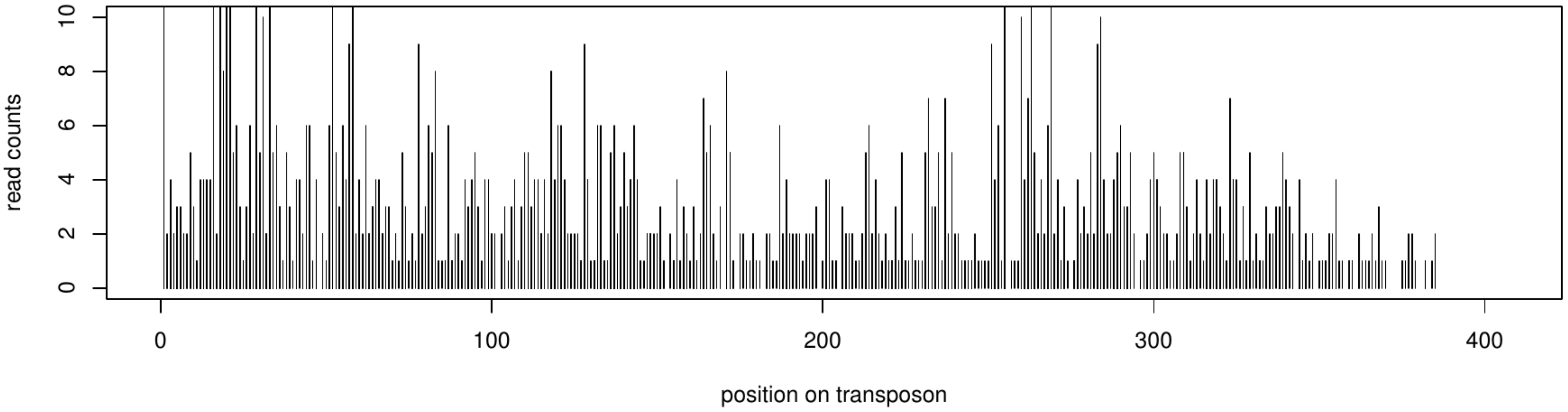

Olat_rnd-5_family-741_LTR/ERVK tg(mitfa:xmrk)

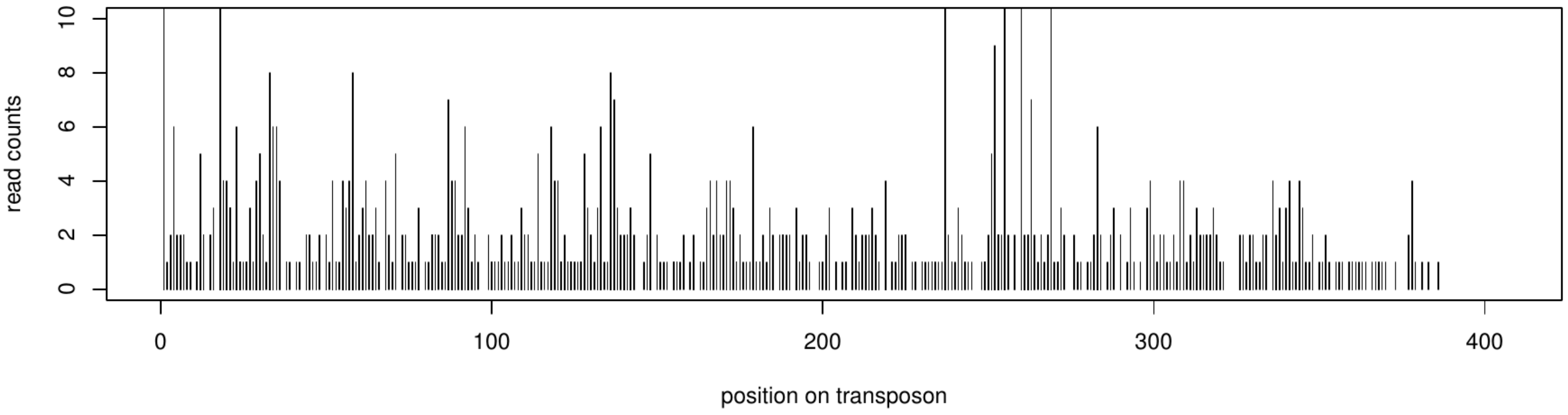

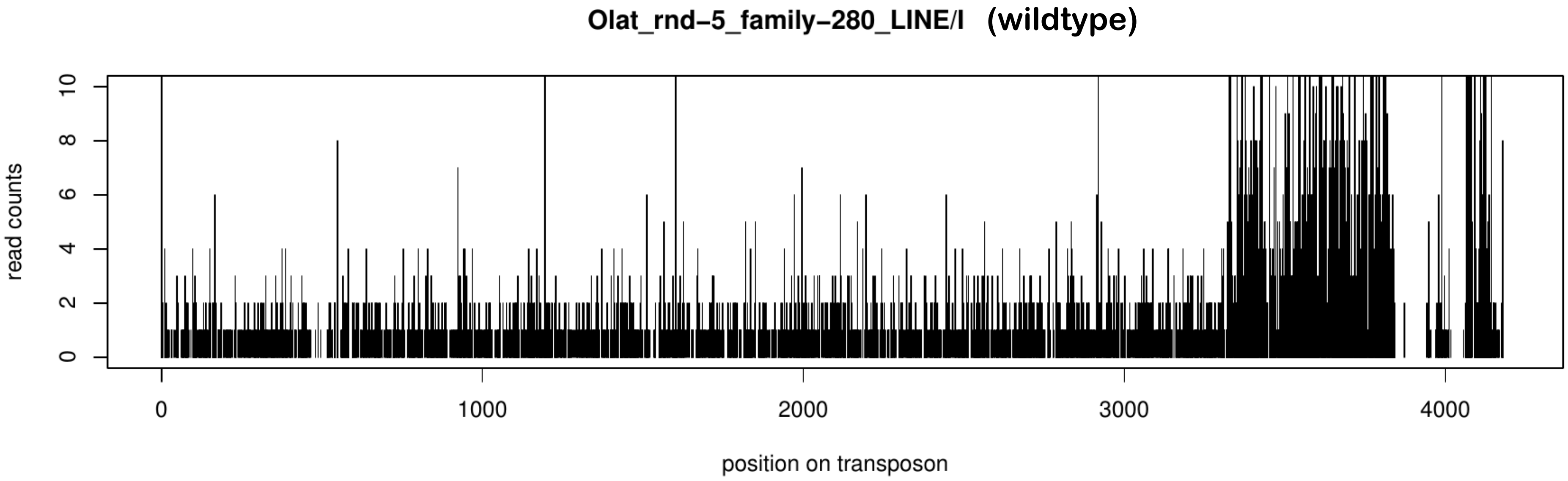
Olat_rnd-5_family-280_LINE/I (wildtype)
read counts
0
2
4
6
8
10
0
1000
2000
3000
4000
position on transposon

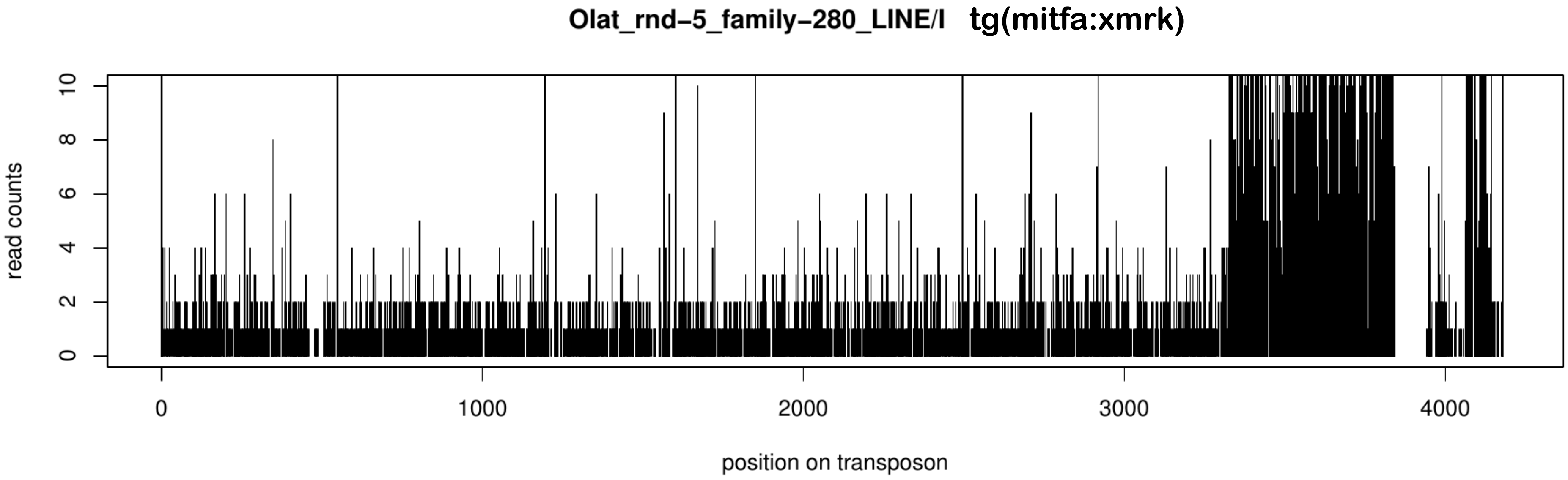
Olat_rnd-5_family-280_LINE/I tg(mitfa:xmrk)
read counts
0
2
4
6
8
10
0
1000
2000
3000
4000
position on transposon

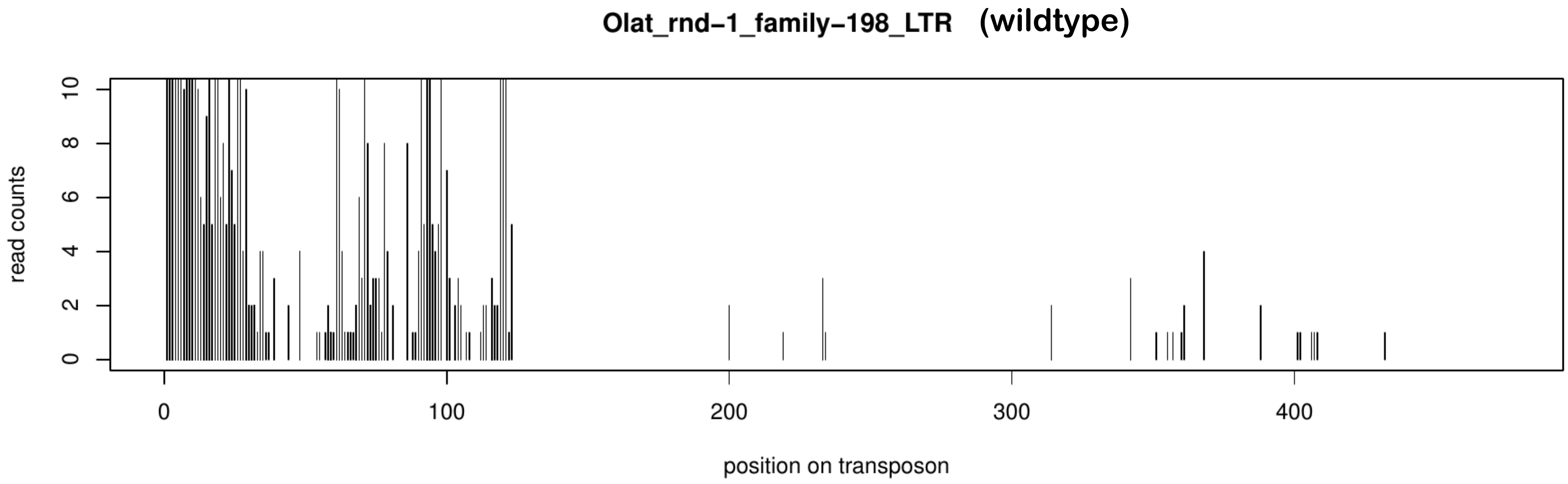
Olat_rnd-1_family-198_LTR (wildtype)
read counts
0
2
4
6
8
10
0
100
200
300
400
position on transposon

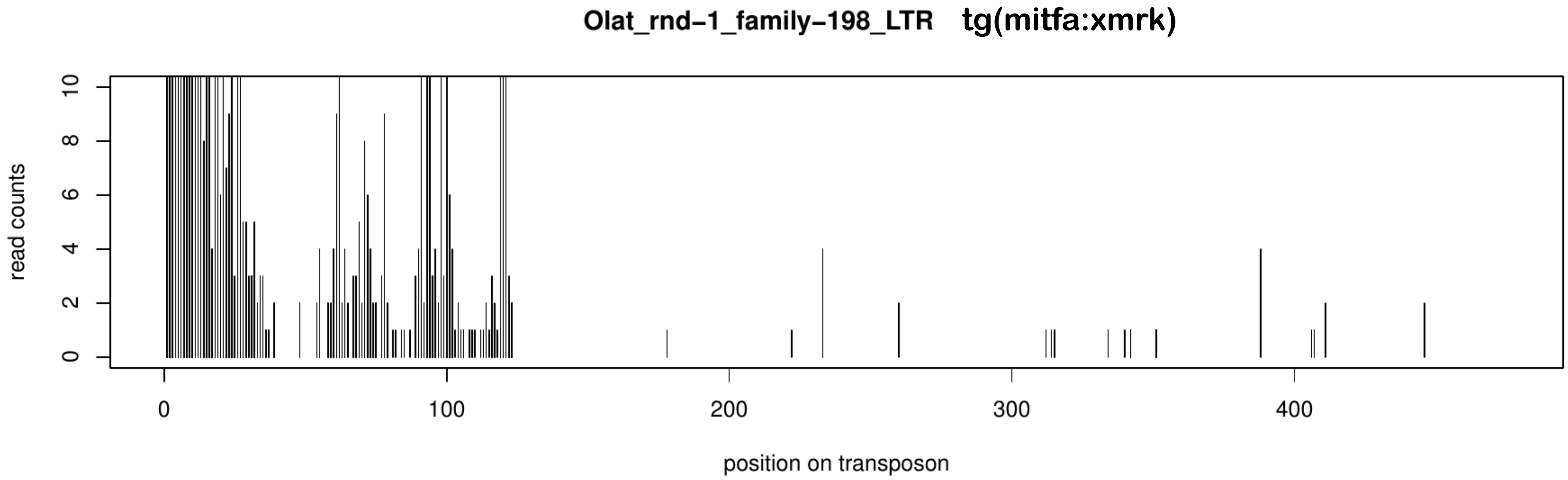
Olat_rnd-1_family-198_LTR tg(mitfa:xmrk)
read counts
0
2
4
6
8
10
0
100
200
300
400
position on transposon

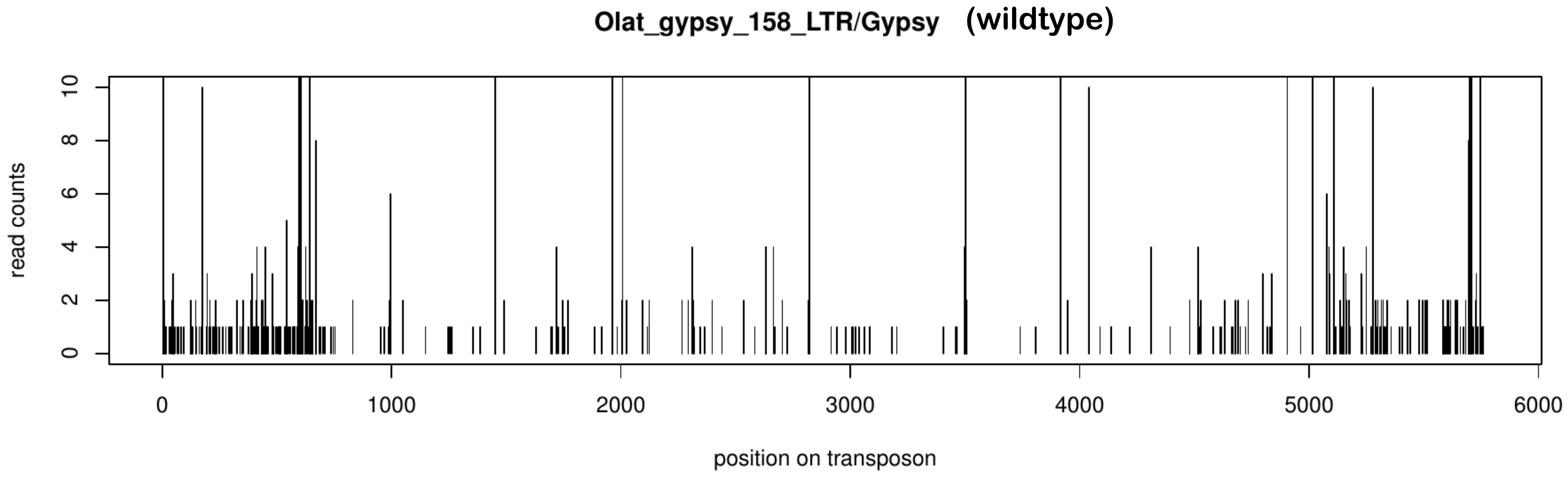

Olat_gypsy_158_LTR/Gypsy (wildtype)
read counts
position on transposon
0
2
4
6
8
10
0
1000
2000
3000
4000
5000
6000

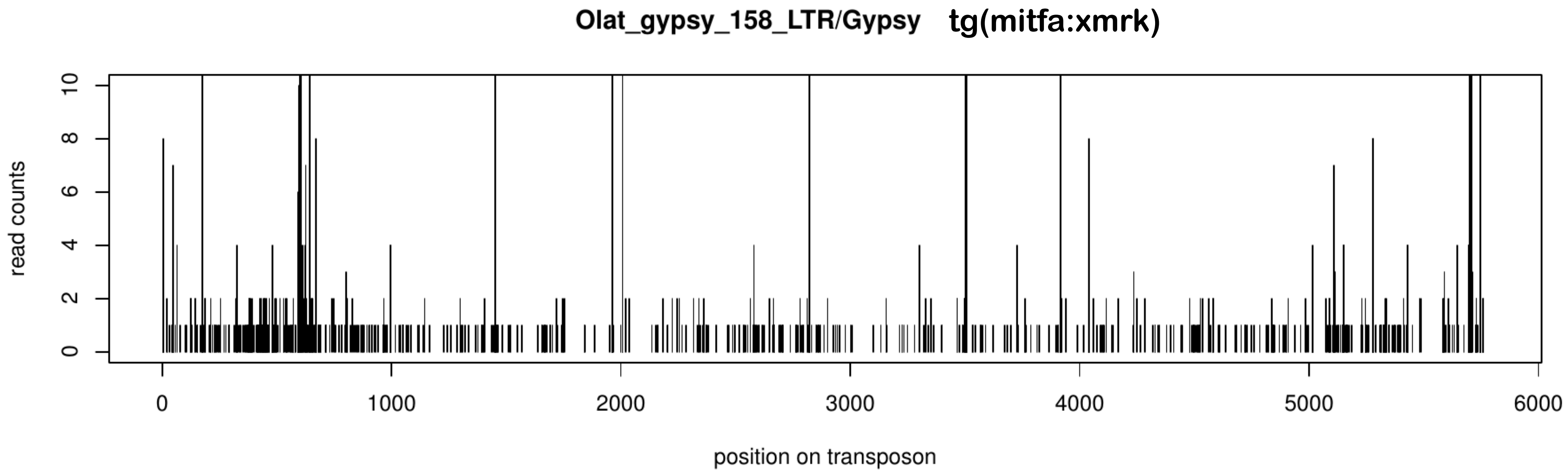

Olat_gypsy_158_LTR/Gypsy tg(mitfa:xmrk)
read counts
position on transposon
0
2
4
6
8
10
0
1000
2000
3000
4000
5000
6000

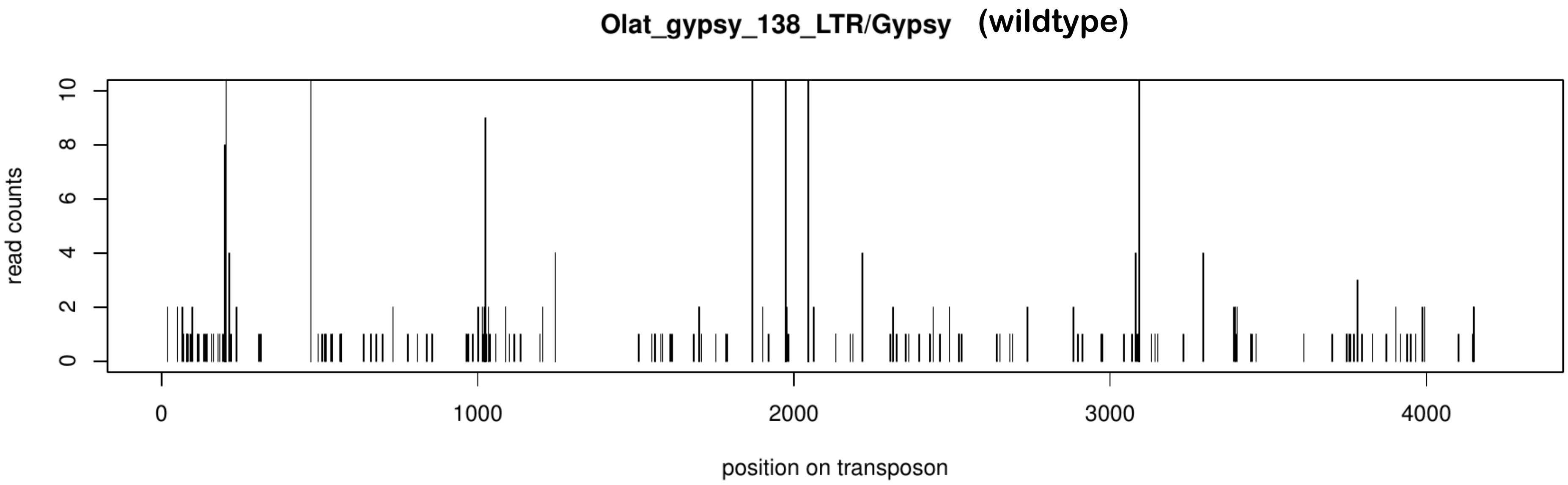
Olat_gypsy_138_LTR/Gypsy (wildtype)
read counts
0
2
4
6
8
10
0
1000
2000
3000
4000
position on transposon

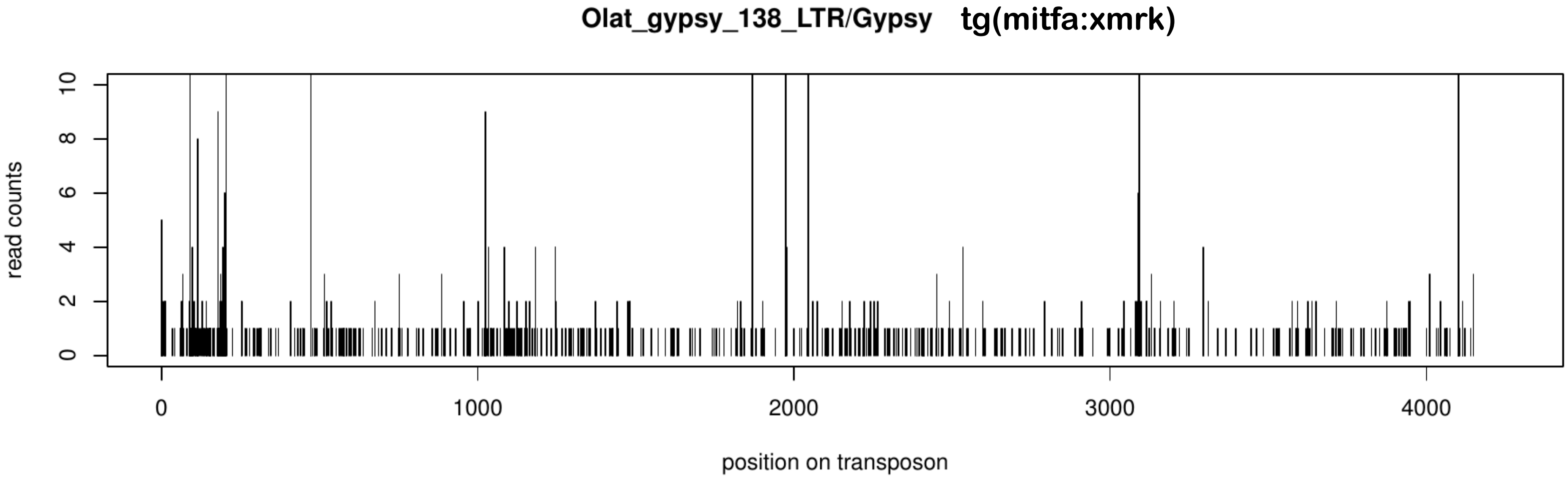
Olat_gypsy_138_LTR/Gypsy tg(mitfa:xmrk)
read counts
0
2
4
6
8
10
0
1000
2000
3000
4000
position on transposon

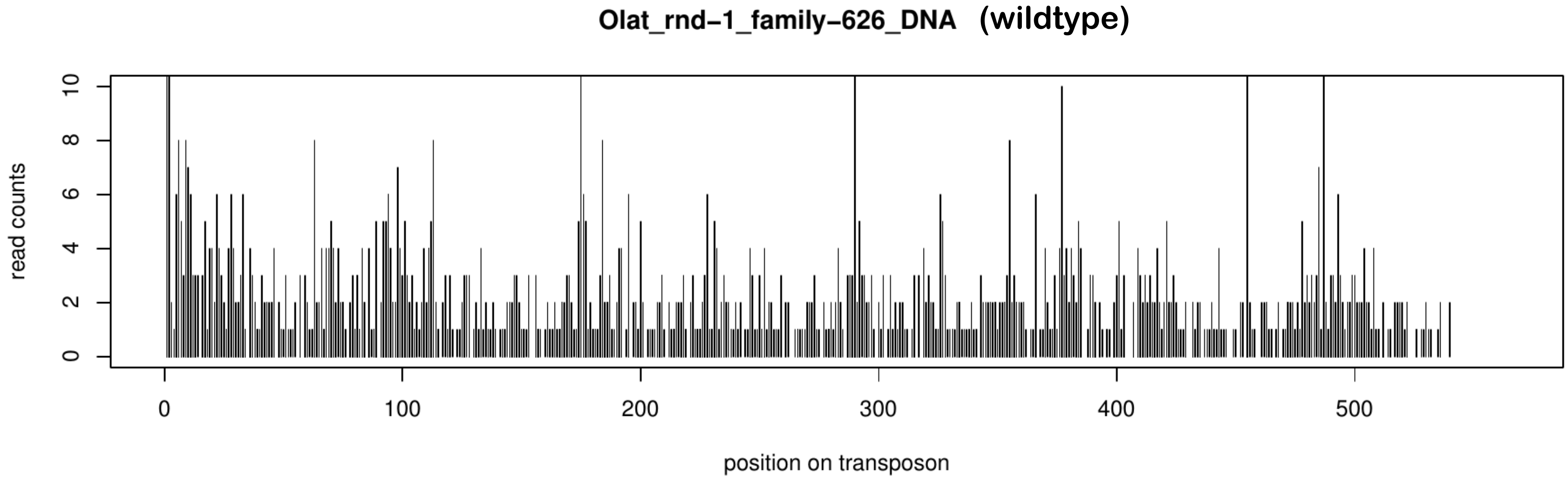
Olat_rnd-1_family-626_DNA (wildtype)
read counts
0
2
4
6
8
10
0
100
200
300
400
500
position on transposon

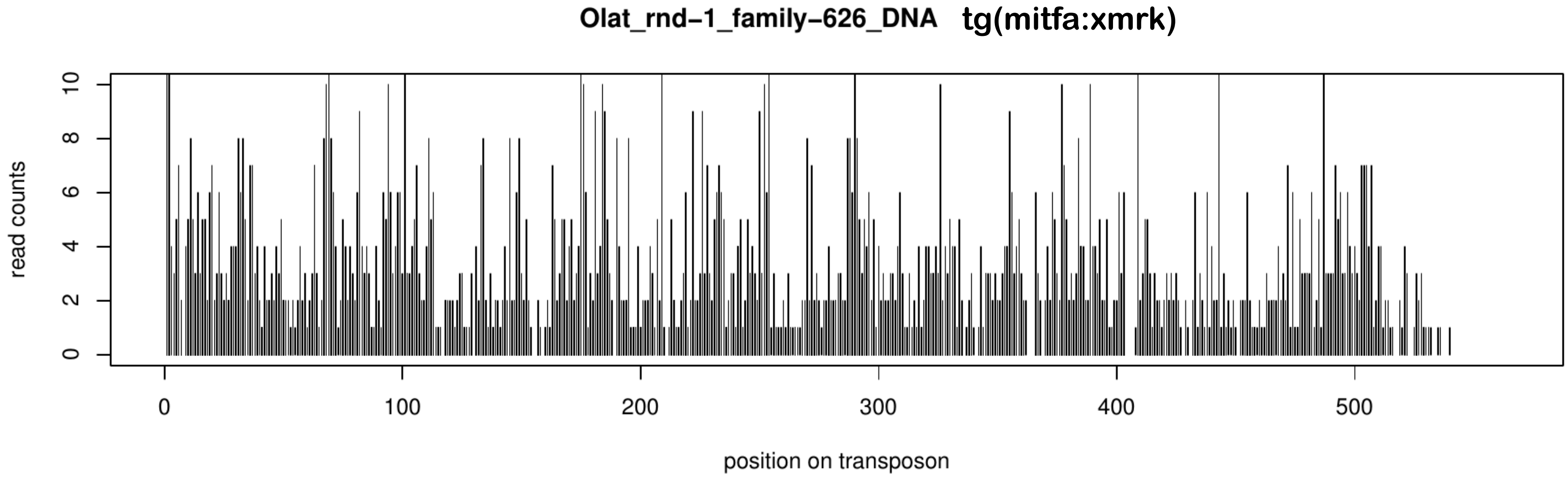
Olat_rnd-1_family-626_DNA tg(mitfa:xmrk)
read counts
0
2
4
6
8
10
0
100
200
300
400
500
position on transposon

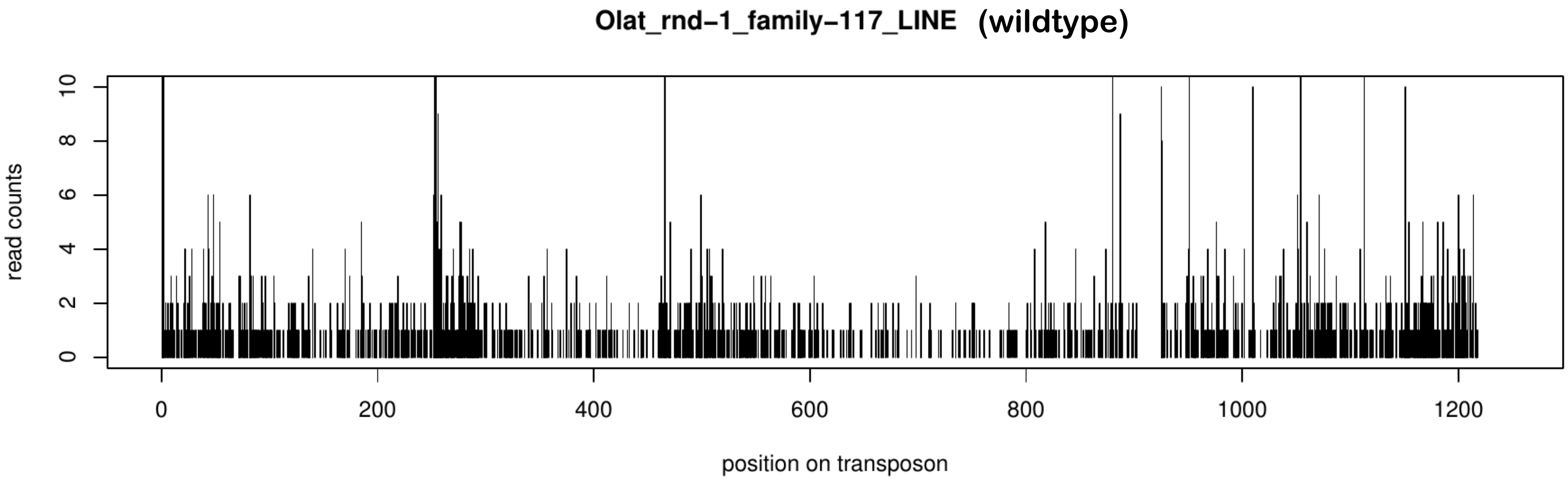
Olat_rnd-1_family-117_LINE (wildtype)
read counts
0
2
4
6
8
10
0
200
400
600
800
1000
1200
position on transposon

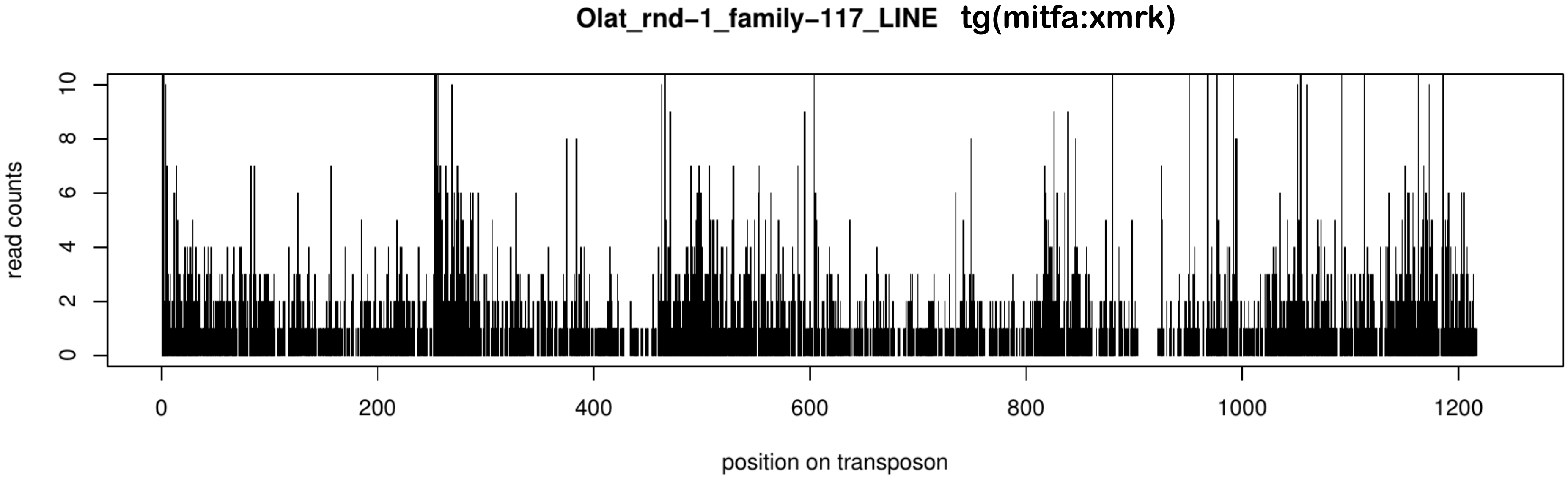
Olat_rnd-1_family-117_LINE tg(mitfa:xmrk)
read counts
0
2
4
6
8
10
0
200
400
600
800
1000
1200
position on transposon
